# Light from Afield: Fast, High-Resolution, and Layer-Free Deep Vat 3D Printing: Focus Review

**DOI:** 10.1021/acs.chemrev.4c00134

**Published:** 2024-07-05

**Authors:** Parth Chansoria, Riccardo Rizzo, Dominic Rütsche, Hao Liu, Paul Delrot, Marcy Zenobi-Wong

**Affiliations:** † Department of Health Sciences and Technology, ETH Zürich, Zürich 8093, Switzerland; ‡ John A. Paulson School of Engineering and Applied Sciences, Harvard University, Boston, Massachusetts 02134, United States; § Wyss Institute for Biologically Inspired Engineering, Harvard University, Boston, Massachusetts 02215, United States; ∥ Department of Bioengineering, Stanford University, Stanford, California 94305, United States; ⊥ Basic Science & Engineering (BASE) Initiative, Stanford University School of Medicine, Stanford, California 94305, United States; # Readily3D SA, EPFL Innovation Park, Lausanne 1015, Switzerland

## Abstract

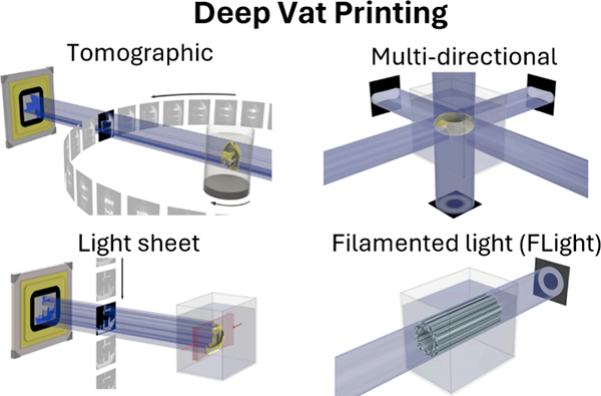

Harnessing light for cross-linking of photoresponsive materials has revolutionized the field of 3D printing. A wide variety of techniques leveraging broad-spectrum light shaping have been introduced as a way to achieve fast and high-resolution printing, with applications ranging from simple prototypes to biomimetic engineered tissues for regenerative medicine. Conventional light-based printing techniques use cross-linking of material in a layer-by-layer fashion to produce complex parts. Only recently, new techniques have emerged which deploy multidirection, tomographic, light-sheet or filamented light-based image projections deep into the volume of resin-filled vat for photoinitiation and cross-linking. These Deep Vat printing (DVP) approaches alleviate the need for layer-wise printing and enable unprecedented fabrication speeds (within a few seconds) with high resolution (>10 μm). Here, we elucidate the physics and chemistry of these processes, their commonalities and differences, as well as their emerging applications in biomedical and non-biomedical fields. Importantly, we highlight their limitations, and future scope of research that will improve the scalability and applicability of these DVP techniques in a wide variety of engineering and regenerative medicine applications.

## 1 Introduction: Deep Vat Printing (DVP)

3D printing or additive manufacturing has transformed the modern manufacturing landscape, redefining how objects are conceptualized, designed, and produced. The approach has quickly transitioned beyond the enabling technology for prototyping, to creating functional components for virtually all manufacturing sectors (aerospace, biomedical, semiconductor, etc.).^1^ Toward a sustainable future, 3D printing reduces material waste and can consume less energy compared to conventional processes such as milling and drilling. Amid this transformative wave, light-based printing techniques have harnessed the power of precise light projection to selectively cross-link liquid photopolymers, introducing unprecedented levels of precision to the 3D printing process.^2^ These techniques offer a broad range of resolution, speed and geometric complexities that were previously unattainable through droplet deposition or material extrusion.

**Figure 1:**
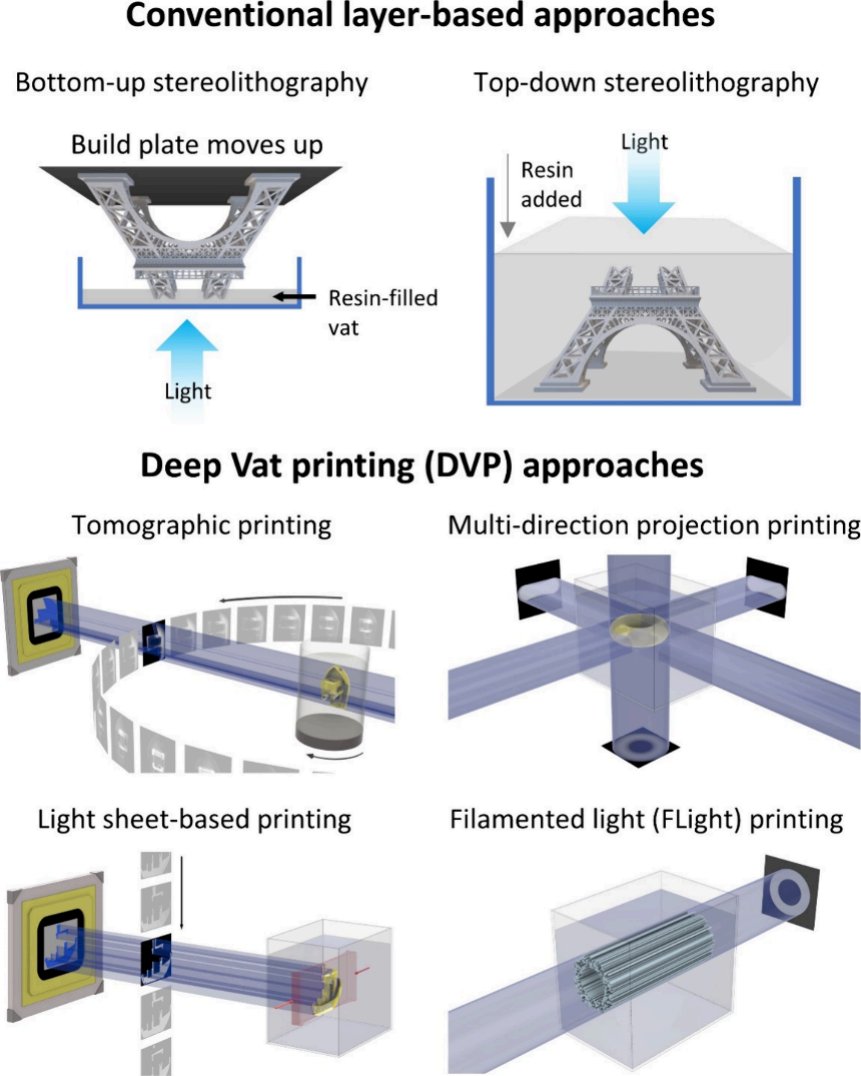
Conventional layer-by-layer processes and DVP approaches. Detailed figures on the working principles of each DVP approach are provided in Section 2.

The most widely used light-based printing methods utilize the layer-by-layer printing approach (Figure 1), where the material is cross-linked by traversing a laser to induce point-wise cross-linking (e.g., sterolithography (SLA)^3^ or two-photon polymerization (2PP)^4^) or the entire layer is cross-linked at once using image projection (e.g., digital light projection (DLP) deploying digital micromirror devices (DMDs) or Liquid Crystal Displays (LCDs)).^5,6^ In these approaches, a thin layer of material is photopolymerized through light projection, where it attaches to the already cross-linked material of the previous layer. Photoabsorbers are often deployed to prevent light from penetrating into the cross-linked layers and causing overpolymerization.^5^ In contrast to SLA or DLP printing, 2PP can offer nanoscale resolution.^7,8^ This is made possible through the simultaneous absorption of two photons, which allows a highly localized energy deposition for cross-linking. Through improved laser light focusing and guidance techniques, printing rates with 2PP (up to 450 mm^3^/h) are rapidly catching up to the more conventional SLA or DLP process. Utilizing light as the sculpting tool can, with certain processes such as continuous liquid interface production (CLIP), expedite the manufacturing by speeding-up the transition between the layers in the printing process.^9,10^ Faster production cycles make it particularly valuable for rapid prototyping and iterative design processes. Moreover, the smoother surface finishes achieved through light-based printing simplify postprocessing, further accelerating production timelines.^11,12^

In the past few years, contrary to the above approaches involving additive cross-linking of thin layers of material, new printing methods have been developed, which rely on light propagation deep into a resin-filled vat to achieve a cumulative light dose for cross-linking of the material into complex shapes. Within the 3D printing or additive manufacturing community, the term “volumetric printing” is frequently used to describe these techniques, but there are discrepancies in the usage of the term. A common understanding is that a volumetric printing approach should generate the entire volume of the 3D printed structure simultaneously through light projection, which would encompass the techniques of multidirection projection^13,14^ or tomographic printing.^15,16^ However, there are other techniques which feature some commonalities in terms of the process principles of light propagation deep into the volume of resin vat, as well as the photoinitiation and cross-linking mechanisms. These include light sheet-based stereolithography^17,18^ or filamented light (FLight) biofabrication.^19,20^ Given the fact that the cross-linking is taking place in a volume of resin-filled vat, the term volumetric printing has also been used for light sheet-based techniques such as Xolography.^18,21^ To circumvent these discrepancies, we instead propose the use of the term “Deep Vat” printing in this review, to be able to cover all four techniques together. This also allows one to distinguish between Deep Vat printing (DVP) methods and the more conventional techniques, where only a small region of the material within the vat is available for photo-cross-linking at a time.

Notably, there have been recently published topical reviews on volumetric printing,^22−24^ and volumetric printing has also been briefly covered in other broader reviews on 3D printing and its applications^25−27^ and benchmarked in terms of its print speed and voxel resolution compared to other printing approaches in some reviews.^28,29^ However, these reviews have focused only on the applications and materials for tomographic printing (a subset of the DVP methods) and have not covered the common principles of DVP approaches. In this review, we highlight the principles behind these printing approaches (Figure 1), their commonalities and differences, and discuss in detail the chemical compositions and cross-linking mechanisms of photoresins compatible with each approach. We then highlight the current state of research in DVP, and some of the important future considerations for achieving high resolution and high fidelity prints with the different approaches and the materials involved therein. Lastly, we discuss the scope for improvement in these techniques when collectively looking at the field of DVP.

## 2 Mechanisms for DVP Approaches

### 2.1 Commonalities in the Process Principles

#### 2.1.1 Gelation Threshold

All these approaches rely on the threshold behavior of photo-cross-linkable polymers, that will solidify, or gel, above a certain cross-linking degree, into a polymer network. For instance, in step growth polymerization, the solidification, or gelation, threshold γ_c_ of a growing polymer network, was statically defined by Flory^30^ as
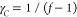
1where *f* is the functionality of the polymer precursor (in other words, *f* is the number of functional groups in a polymer chain of the photoresin). As the photoresin absorbs the light, the cumulative 3D light dose in a specific volume within the resin vat locally exceeds its gelation threshold, thus locally cross-linking it and creating the desired object.

#### 2.1.2 Light Propagation Fundamentals

All DVP approaches deploy at least one collimated light beam, i.e., the light beam has negligible divergence or convergence. Light collimation is ensured at least over the length scale of the resin vat in which it is projected, which lengthens the depth of field and ensures uniformity of the projected images. While the collimated light propagates into the photoresin, it undergoes attenuation which can generally be defined using the Beer–Lambert law:
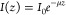
2where *I(z)* is the intensity (W/cm^2^) at depth *z* (cm) in the photoresin, *I*_*0*_ is the incident intensity in the photoresin and μ (cm^–1^) is the attenuation coefficient of the resin. The attenuation coefficient μ is the sum of the absorption coefficient μ_a_ and scattering coefficient μ_s_. The absorption coefficient μ_a_ defined as
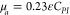
3where *C*_*PI*_ is the molar concentration and ε is the molar absorptivity (M^–1^·cm^–1^) of the photoinitiator. As an example, for lithium phenyl-2,4,6-trimethylbenzoylphosphinate (LAP) photoinitiator with molar absorptivity of 218 M^–1^·cm^–1^ at 365 nm and a concentration of 5 mM,^31^ the attenuation coefficient is approximately 0.25 cm^–1^. To consider the contribution of μ_s_, one has to consider the presence or absence of scattering particles (e.g., cells or other micro/nanoparticles). For pure monomer resins which do not have scattering particles, the size of the monomers is less than a tenth of the wavelength. In these cases, the light undergoes Rayleigh scattering, where the scattering coefficient can be expressed using the Smoluchowski–Cabannes formula:^18,32^
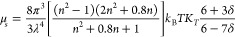
4Where λ is the wavelength, *n* is the refractive index of the resin, *k*_*B*_ is the Boltzmann constant, *T* is the temperature during printing, *K*_*T*_ is the isothermal compressibility (Pa^–1^) of the material, and δ is the depolarization ratio. As an example, for a pentaerythritol tetraacrylate (PETA)-based resin^18^ with refractive index *n* = 1.56, *K*_*T*_ = 5.7 × 10^–10^ Pa^–1^, δ = 0.4, exposed to 365 nm wavelength at room temperature (T = 293 K), the scattering coefficient would be approximately μ_S_ = 7.6 × 10^–4^ cm^–1^. Therefore, in most cases for pure resins (i.e., without any added particles or cells), the scattering coefficient is significantly lower than the absorption coefficient and can often be neglected.^18,33^ Therefore, considering only the absorption coefficient of 0.25 cm^–1^, the intensity at a distance of 2 cm (typical size of printing vials in DVP techniques) into the resin container is approximately 60% of the intensity projected into the resin (as per eq 2). Of note, for highly cellular or particle-laden resins, light scattering cannot be ignored, yet it cannot be theoretically determined. In this approach, performing actual measurements of light attenuation in the resin and digitally compensating for the light intensity could enable deeper light penetration and successful printing^34,35^ (discussed in Section 4).

#### 2.1.3 Photo-Cross-Linking Kinetics

The characteristic time scale, t_c_, of a printing process can be compared to the characteristic length of the different physicochemical phenomena at play, to assess which phenomena are relevant during a DVP process. For instance, photo-cross-linking is a highly exothermic reaction, that can, because of Arrhenius kinetics, lead to local autocatalysis that follows the diffusion of the cross-linking-induced thermal front.^36^ The effect of heat on the photo-cross-linking kinetics can be represented as.^36^
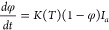
5where φ is the local fraction of conversion of the monomer into a polymer, *K*(*T*) is the temperature dependent rate constant of cross-linking, (1−φ) is the local fraction of remaining monomer, *I*_*a*_ is the local absorbed light intensity. The local conversion fraction φ = 0 at the initiation of cross-linking and φ = 1 when fully polymerized. In the case that the temperature increase *ΔT* (in Kelvin) is small compared to the experimental initial temperature *T*_*0*_, a simplified nondimensional Arrhenius law for the temperature-dependency of the cross-linking rate constant *K(T)* is^36^
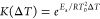
6where *R* is the gas constant in J·K^–1^·mol^–1^ and *E*_*a*_ the cross-linking activation energy in J·mol^–1^. To get a numerical sense of this equation, the cross-linking rate constant K locally doubles when the cross-linking-induced heat locally increases the temperature by 30K, with a cross-linking activation energy of *E*_*a*_ = 20 kJ/mol^37^ and an initial temperature *T*_*0*_ = 298 K.

Further estimation of the thermal diffusion of this cross-linking-induced autocatalysis helps to assess if it could reduce the printing resolution. More specifically, the 3D thermal front induced by the 3D light dose deposited in the photopolymer vat could diffuse during the printing process and cause undesired cross-linking in the resin vat. The characteristic thermal diffusion length *L*_*t*_ of the heat front generated during a photo-cross-linking process of characteristic time scale (*t*_*c*_) follows Fick’s law:
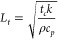
7where *k* is the thermal conductivity (in W·m^–1^·K^–1^) and *ρc*_*p*_ the volumetric heat capacity of the resin (J·m^–3^·K^–1^). eq 7 yields a significant thermal diffusion length of *L*_*t*_ = 1.9 mm, assuming that the characteristic time scale *t*_*c*_ of a standard printing is 30 s, the photoresin thermal conductivity is *k* = 0.2 W·m^–1^·K^–1^ and the volumetric heat capacity is *ρc*_*p*_ = 16 MJ·m^–3^·K^–1^ (for poly(methyl methacrylate) (PMMA) resin^38^).

Similarly, the diffusion length *L*_*d*_ of reactive and inhibiting species, such as radicals and oxygen, during a printing process of characteristic time scale *t*_*c*_ can be defined using Fick’s law:^39^
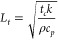
8where *D* is the diffusion coefficient of the reactive or inhibiting species. Interestingly, Stokes–Einstein equation shows that the diffusion coefficient *D* inversely scales with the photoresin dynamic viscosity η:
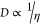
9

Hence, knowing the dynamic viscosity of a photoresin and the characteristic time scale of the printing process, one can theoretically predict if the diffusion length of reactive or inhibiting species could affect the resolution of the printed object. Numerically, in a 10 Pa·s viscous resin, the diffusion coefficient of oxygen, which acts as a radical scavenger, is 1.2 × 10^–13^ m^2^/s. The diffusion length is less than 4 μm for a printing time scale of *t*_*c*_ of 30 s, and the oxygen diffusion blurring of the dose distribution can be ruled out for such viscous resins.

### 2.2 Multidirection Projection and Tomographic Printing

**Figure 2:**
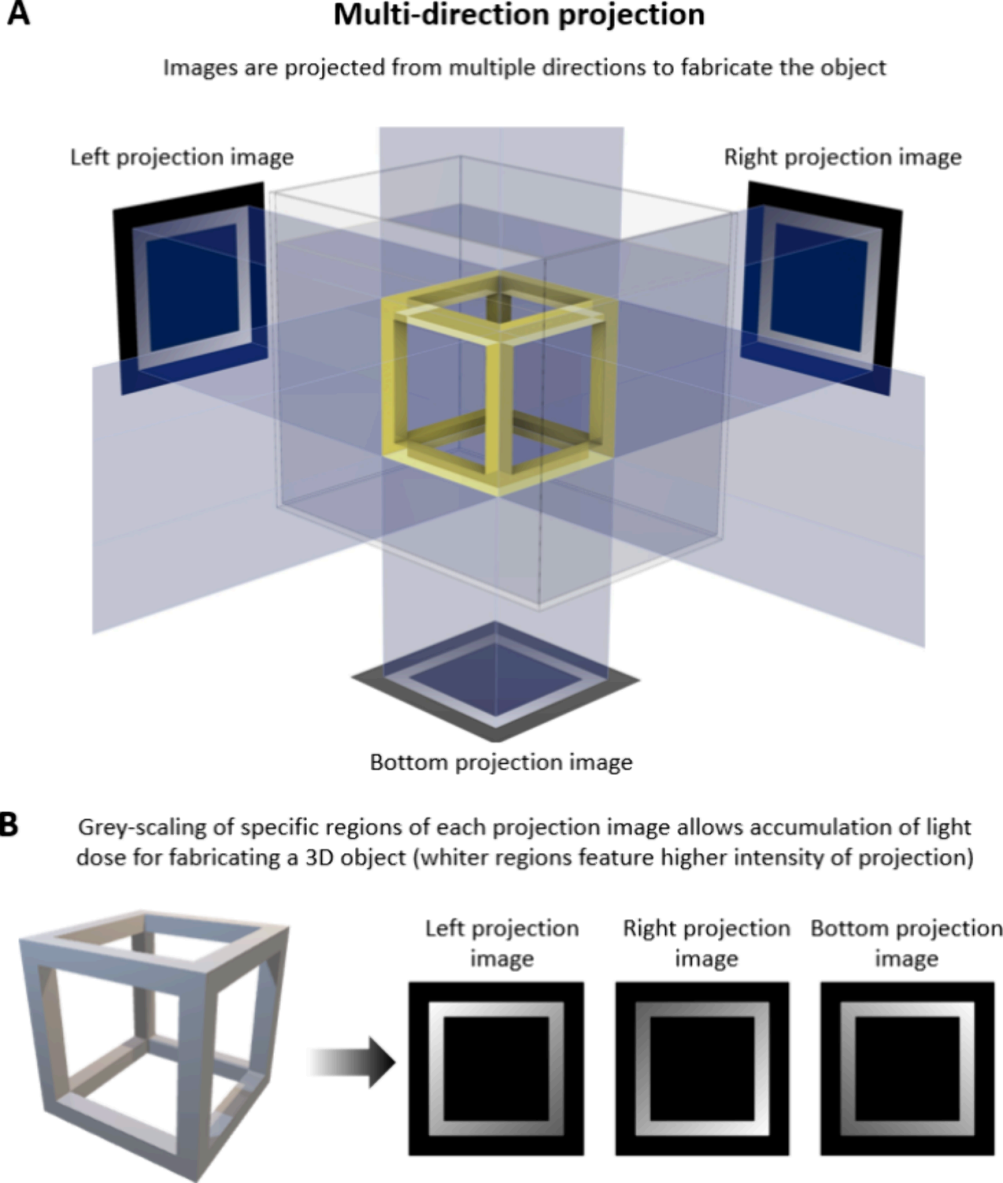
A. DVP approach utilizing accumulation of light dose from multiple directions in the photoresin. B. The vial is kept static, and different images featuring gray scale gradient of intensity patterns are projected from two or three different directions to cross-link the resin.^14^

**Figure 3:**
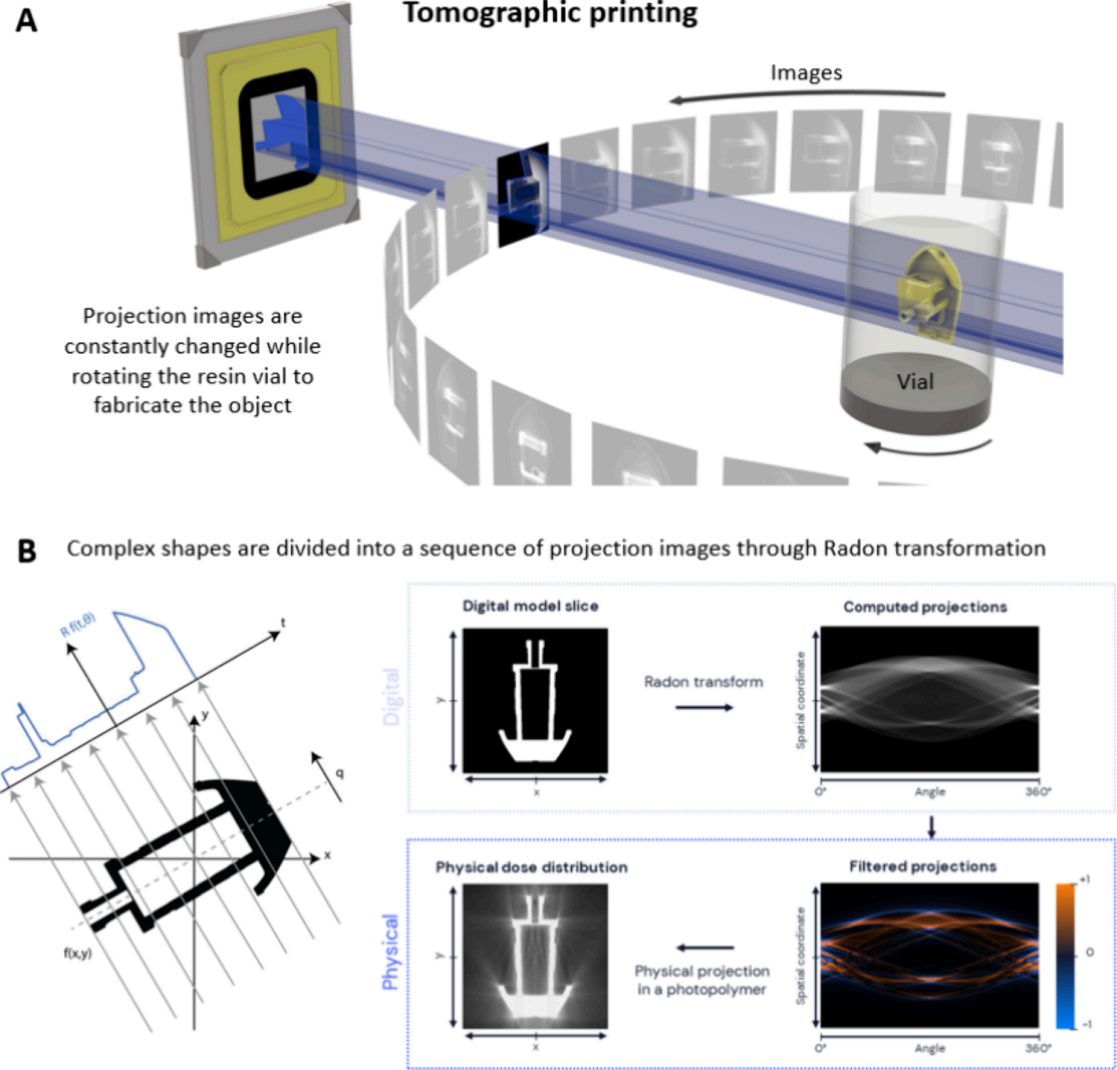
Tomographic printing. A. Projection images are changed with every step of the vial rotation to achieve a cumulative light dose to fabricate the 3D object. The model 3DBenchy by Creative-Tools.com is licensed under a Creative Commons Attribution-No Derivatives 4.0 International License. B. Tomographic reconstruction of the object can be performed through a radon transformation, where, based on the rotation angle (θ) of the object with respect to the light projection and its spatial coordinate (q) and lateral shift (t), the projection images are derived for each rotation. An overlap of different images projected from a single direction but synchronized with vial rotations achieves the cumulative light dose to cross-link the object.^33^

Tomographic printing relies on the generation of a cumulative 3D light dose distribution in resin-filled vats, which will locally trigger the photo-cross-linking reaction. To achieve this cumulative 3D light dose, the 2D patterns of light are projected either from multiple directions (Figure 2),^14,40^ or by changing the projection images within a rotating resin-filled vat (Figure 3).^41,42^ One of the seminal works in domain of multidirection projection printing was by Shusteff and colleagues,^14^ who used beam projections from three directions to define a 3D light dose distribution within a cuboidal container of photoresin (Figure 2A), thereby locally curing the photoresin and creating a 3D object. As with other DVP approaches, at any depth z, the absorbed irradiance per unit volume is governed by Beer–Lambert Law (eq 2). Accordingly, the projection image for each direction features intensity gradients (executed by gray-scaling of the images; whiter regions feature higher intensities, Figure 2B) to be able to compensate for the reduced intensity at depth z due to absorption by the photoinitiator.^14^ The complexity of the 3D light dose distribution and the subsequent 3D printed objects that can be generated via multidirection projection approach is limited by the number of projection directions through which light can be absorbed and integrated, as well as the depth of focus of the projected beams. As such, in this multidirection projection approach, achieving complex shapes such as helices or perfusable loops may need dynamic focusing, which would require nuanced optical focusing and image projection.^14,40^

Dramatically increasing the number of projection directions using a tomographic approach has been an evolution from the multidirection projection approaches, to be able to create more complex 3D light dose integrals and distributions within the photoresin volume. In tomographic printing (Figure 3A), the sequence of light patterns needed to create the 3D object is computed using the Radon transform, which relates an object to its projections, and its inverse function, which relates the back-projections with the reconstruction of the object.^33,41^ The Radon transform *Rf(t,θ)* is described in eq 10 and in Figure 3B:
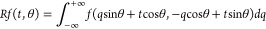
10where θ represents the projection angle, *q* is the spatial coordinate over which the line integral of the 2D object map is computed (in other words, the direction of projection), and *t* represents the lateral shift at which each line integral is computed.

In tomographic printing, the sequence of 2D light patterns to print a 3D object is derived by first converting a digital 3D model of the object, like an STL format file, into a three-dimensional voxel map, which corresponds to a 3D matrix of “1” and “0” that respectively indicate the presence and the absence of the object at each location in space, in the case of a binary object. In the case of an 8-bit grayscale object, the 3D matrix will have values ranging from “255” to “0” for the highest and lowest cross-linking degree of the 3D object, respectively. Next, individual 2D sections of the object’s 3D matrix are derived, followed by calculating 1D projections over 360° using Radon transform (Figure 3B). The discrete set of angles along which the 1D projections are calculated should ideally satisfy the Nyquist-Shannon sampling theorem to match the intended printing voxel size. Subsequently these 1D projections are filtered with a Ram-Lak filter, which yields 2D projections with both negative and positive values.^43^ Theoretically, such 1D projections would cumulatively result in a perfect reconstruction of the object when back-projected into a volume, but negative values of light cannot be physically produced. Thus, a positive threshold is applied on these 1D projections to remove the negative values. For each projection angle, the 1D projections are combined into the 2D image that will be back-projected with light onto the resin-filled vat, to create the 3D light dose distribution that matches the 3D object shape.

### 2.3 Filamented Light (FLight) Printing

**Figure 4:**
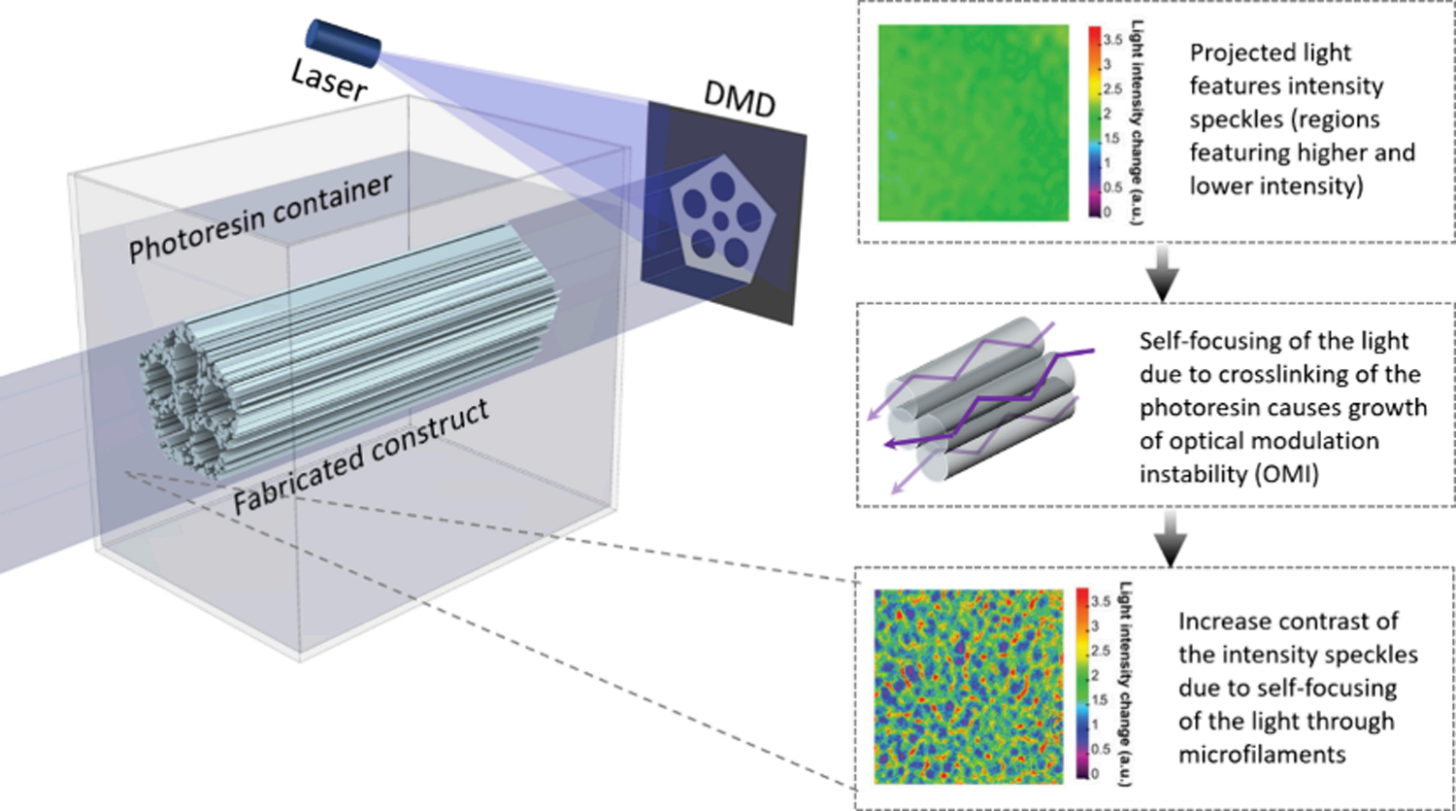
FLight projection: A DVP approach based on self-focusing of speckled light within the resin-filled vat to fabricate constructs featuring aligned filaments^44^ (adapted from ref^44^ copyright 2022 Wiley under CC-BY 4.0 [https://creativecommons.org/licenses/by/4.0/]).

Multidirection projection and tomographic printing rely on locally exceeding the resin gelation threshold within the volume of photoresin, either through static projections or by dynamic projections from multiple angles. In contrast, FLight relies on the initiation of photo-cross-linking at the interface where the light is first incident on the photoresin and the subsequent propagation into the bulk volume of the photoresin through the optical self-focusing of the light^20,44^ (Figure 4).

As the material cross-links, there is a change in the refractive index (*Δn*) of the material (also known as Kerr effect^45−47^):
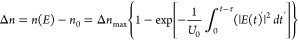
11where *n*_0_ is the initial refractive index, *E(t)* is the instantaneous electric field of irradiated light, Δ*n*_max_ is the maximum attainable change in refractive index (i.e., between fully cured and uncured photoresin), *U*_*0*_ is the critical energy density to initiate cross-linking (units of J/cm^2^), τ is the monomer radical lifetime. One important feature of this approach is the use of monochromatic light sources such as lasers which exhibit spatiotemporal coherence (i.e., the photons share the same frequency and phase). The interference of coherent light with rough surfaces (on the scale of optical wavelength) or with diffusive media generates intensity speckles (also known as a speckle pattern, where the light features several regions of intensity maxima and minima) due to the constructive interference of multiple monochromatic wavefronts. The interaction of a coherent light with media that undergoes refractive index changes in response to light (i.e., optical nonlinearity) is governed by the paraxial wave equation:^47^
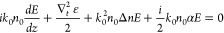
12where *E* is the electric field amplitude, α is the attenuation coefficient of the medium, *z* is the direction of light propagation, and *k*_0_ is the free space wave vector. In the equation above, while the transverse Laplacian operator ∇_t_^2^ = ∂^2^/∂x^2^ + ∂^2^/∂y^2^ highlights the tendency of the light to diverge orthogonally as it propagates through the resin, the beam divergence is counteracted by self-focusing due to the refractive index change (*Δn*) of the resin along the light path. For most photopolymerizable resin formulations, *Δn* is substantial enough to cause a self-trapping of the light due to a total internal reflection (i.e., the cross-linked resin acts like a fiber core with higher refractive index, and the un-cross-linked region acts like the fiber cladding with lower refractive index). As an example, in gelatin-based resins, *Δn* is usually between 0.003 and 0.006.^44,48,49^ In this nonlinear dynamic system, there exists a positive feedback loop between light intensity and the rate of photo-cross-linking. The optical self-focusing of light amplifies the local irradiation dosage further elevating photoinitiation and cross-linking, thereby leading to further self-focusing and completing a positive feedback loop known as optical autocatalysis.^50^

Formation of self-guided solitons due to the optical autocatalysis within cross-linked photopolymers gives rise to light filamentation. This phenomenon has been used to create aligned fibrous structures,^46,50^ where the length of the polymer fiber created has been found to be proportional to the duration of light exposure. Of note, the fiber length can also be affected by the wavelength of the light and the cross-linking chemistry, which is discussed in Section 6. For cases where the light is expanded and shaped into an image (with a digital micromirror device (DMD) or spatial light modulator (SLM)) and then projected into the photoresin, the resulting light filamentation pattern occurs across the entire projected image. Here, across the projected image, the photoresin cross-links first in the regions where the intensity is higher in the speckle pattern. Since there are multiple intensity maxima in the speckle pattern of the projected image, the optical autocatalysis of the light from each of the intensity maxima leads to multiple wavefronts along the cross-linked photoresin (i.e., light propagates across multiple microfilaments in the photoresin). The complete process of filamentation of the light into several microfilaments within a photoresponsive matrix is called optical modulation instability (OMI).^51,52^ The growth of OMI leading to the filamentation of photo-cross-linkable materials has been known in photonics literature,^51,52^ but its applications were underexplored. Notably, due to free radical diffusion and close proximity of the microfilaments in the cross-linked constructs, there are regions where there is slight cross-linking between the individual microfilaments. This holds the individual microfilaments together, resulting in an aligned filamented 3D construct which can be manipulated. The aligned structures created through FLight printing have found promising applications in engineering of anisotropic tissues such as muscle, tendon, and cartilage, which have been discussed in Section 5.

### 2.4 Light Sheet-Based Printing

**Figure 5:**
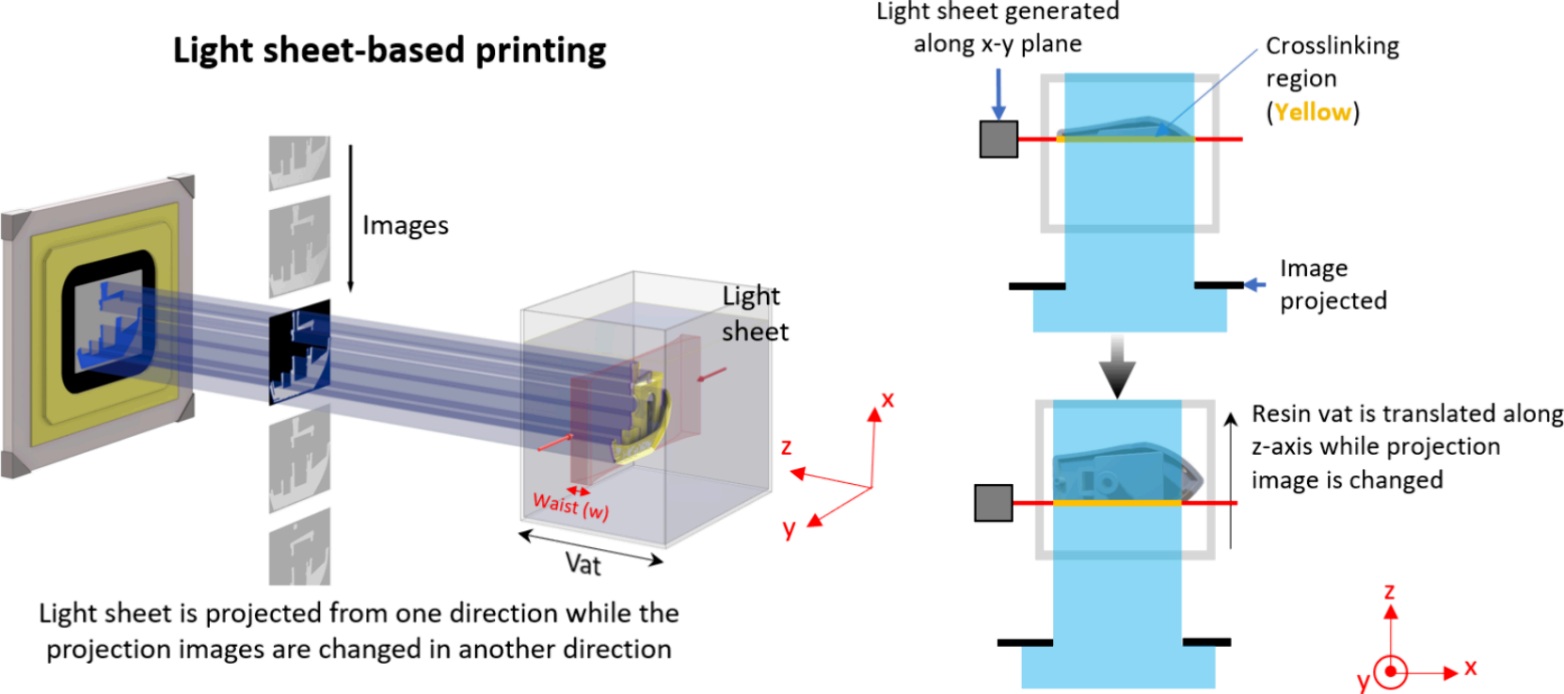
Light sheet-based printing technique which achieves cumulative light dose for photo-cross-linking at the intersection of a light sheet and a projection image. The projection image changes as the light sheet translates along the resin vat, creating the 3D object. Notably, there are subsets of this technique where the light sheet and the projection image feature different wavelengths.^18^

Light sheet-based printing approaches is another DVP approach, where photo-cross-linking is achieved via the intersection of an image projection from one side and a planar light sheet orthogonal to the image projection. Here, the wavelength of the light for the image projection and the light sheet could either be the same (i.e., cross-linking achieved through a cumulative light dose within the resin^17,53^) or different (i.e., cross-linking is executed through free radicals generated from photoswitchable free radical initiators,^18,54^Figure 5).

We have discussed the chemistry of the materials in the subsequent section, but the process mechanics largely depends on the attenuation of the light intensity in the 2D projection image (governed by the Beer–Lambert Law (eqs 2–4)) and the limit of resolution across the depth of the material governed by the width of the light sheet.^18,54^ Determining the optimal size of the light sheet waist (illustrated in Figure 5) with respect to volume depth involves investigating how the width of the light sheet affects printing resolution in the *z*-direction. The horizontal intensity distribution of the light sheet follows a Gaussian function, making Gaussian beam theory suitable for modeling how the beam width changes as it propagates. The optimal width of the light sheet (*w*_0_) at a distance *x* within the resin is expressed as
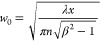
13where λ is the wavelength of the light sheet, *n* is the refractive index of the material at that wavelength, and β is the permitted waist size at a distance of *x*. For example, at the center of a resin container of width D = 30 mm (i.e., *x* = *D*/2 = 15 mm), if the light sheet is permitted to have a 10% increase in the waist size (i.e., β = 1.1), and the resin exhibits a refractive index of 1.56 at 375 nm,^18^ the waist size is approximately 49 μm.

Since the intensity of light reduces further into the depth of the resin (eqs 2–4), there is a possibility that the cross-linked constructs may have a different degree of cross-linking through the depth of the resin container, with reduced stiffness deeper into the vat. This can be counteracted by graying-out the projection images closer to the projection apparatus and increasing the whiteness for projections deeper into the resin. This allows homogeneous light deposition and therefore homogeneous cross-linking density through the entire construct.

## 3 Photoresins for DVP: Cross-Linking Mechanisms and Photoinitiators

**Figure 6:**
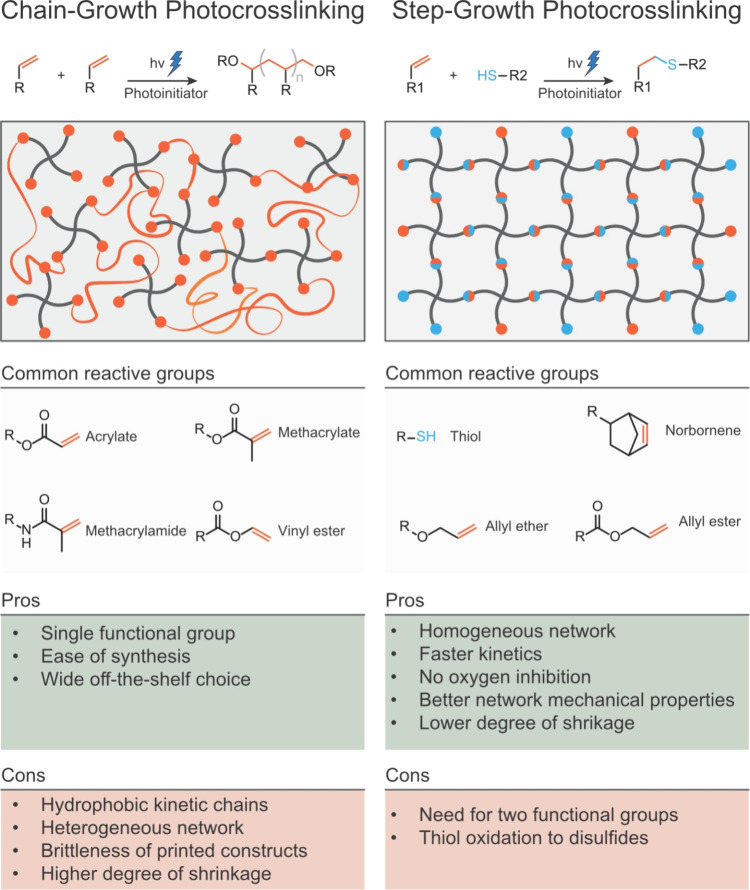
Schematic of chain-growth and step-growth (thiol–ene) mechanisms and resulting networks (top). Common reactive groups for the two mechanisms (middle) and list of pros and cons (bottom).

Photoresins commonly used in DVP approaches are composed of monomers or polymers bearing reactive groups and a light-absorbing photoinitiator. Upon light absorption, the photoinitiator generates radical-initiating species (free radicals) that trigger cross-linking reactions. Such a free-radical based process can proceed via a chain-growth mechanism, a step-growth mechanism, or a mixture of the two based on reactive groups present in the photoresin components (Figure 6). This section offers an overview of the chemistries employed so far in DVP approaches, their characteristics, pros and cons, as well as a glimpse of other, not yet explored, photo-cross-linking approaches.

### 3.1 Photoresins Based on Chain Growth Cross-Linking

The chain-growth radical photo-cross-linking is a mechanism defined by three steps: light dose-triggered radical generation and initiation, propagation, and finally termination.^55,56^ In the first step, the irradiating light beyond a certain threshold dose (mJ/cm^2^) excites the photoinitiator that undergoes photochemical reactions leading to the formation of free radical species (molecule with unpaired electron) that then initiate the cross-linking process. The initiation and propagation of the chain growth mechanism occurs in the presence of carbon–carbon double bonds in the form of vinyl monomers or vinyl-modified polymers. Vinyl functional groups include, in order of higher to lower reactivity, acryloyl, vinyl esters/carbonates, and methacryloyl.^56^ In short, the free radicals undergo radical addition to the double bond of a vinyl monomer forming the propagating site of reactivity in the form of a carbon radical (initiation step). Vinyl monomers then react with the end-chain carbon radical via addition reactions thus extending (growing) the polymer chain (propagation step). The chain-growth cross-linking eventually terminates with the combination of two radical sites, either from propagating chain ends or from a propagating chain end and an initiator radical. Termination can also occur via disproportionation where two propagating chains form separate stable products due to radical induced-hydrogen abstraction. In addition, the end-chain carbon radical can transfer its free radicals to other molecules, such as inhibitors or free-radical scavengers. Propagating radicals are especially vulnerable to molecular oxygen and thus free-radical chain-growth cross-linking is associated with the formation of peroxides. This aspect is of particular importance when considering photoresins for biological applications that require the cross-linking to occur under physiological oxygen conditions and presence of cells sensitive to potentially harmful radicals and reactive oxygen species (ROS).^2,57,58^

Chain growth-based photoresins, particularly those featuring acrylate and methacrylate groups, have been widely used for light-based 3D printing due to the commercial availability of a wide variety of products, from monomers to synthetic and natural polymers, their relatively simple synthesis, and their general ease of use and robustness.^2^ On the other hand, due to the chain-growth mechanism, these photoresins are associated with several generally undesired characteristics. For example, the propagation reaction results in a significant amount of unreacted reactive groups in the final cross-linked network that can represent a source of toxicity or undesired reactions.^59−61^ Also, the formation of the kinetic chain is associated with a considerable network heterogeneity featuring high and low cross-linked domains that cause a high degree of shrinkage of the cross-linked material and thus loss of printing fidelity. In addition, the inhomogeneous chain-growth networks are accompanied by a reduction in mechanical properties, showing more brittle and glassy properties when compared to more homogeneous networks.^62−64^ Other properties, such as sensitivity to oxygen inhibition,^65,66^ can instead be seen as advantageous for specific applications. For example, in CLIP printing,^9^ the oxygen inhibition of chain-growth photoresins is leveraged to form an unreactive interface (dead-zone) in a DLP setup that prevents the resin from adhering to the vat bottom, thus allowing a much faster, continuous printing process. In the context of DVP, oxygen inhibition enhances the nonlinear response of the photoresin to curing illumination and thus contributes to an easier definition of a critical gelation threshold.

### 3.2 Photoresins Based on Step-Growth Cross-Linking

Considering the drawbacks of the chain-growth systems, step-growth photo-cross-linking mechanisms are increasingly being used in light-mediated printing, particularly when related to biomedical applications.^67^ A number of light-triggered step-growth reactions also fall within the definition of click chemistries, thus the name photoclick chemistry has been used.^68,69^ As defined by the seminal work of Sharpless and colleagues,^67^ click reactions are modular, highly efficient and selective reactions that proceed under mild conditions with nontoxic end products. Among these, the thiol–ene reaction has been the most widely used. In this case, the PI-generated radical species are responsible for the formation of thiyl radicals due to hydrogen abstraction from the thiol group. The thiyl radical subsequently attacks the double bond of the alkene (ene) species, thus forming a carbon-centered radical that then abstracts the hydrogen from another thiol forming a new thiyl species. The thiol–ene network formation is insensitive to oxygen inhibition and generally forms significantly faster than chain-growth network. In addition, step-growth mechanisms produce more homogeneous and predictable networks with reduced shrinkage behavior and significantly better mechanical properties (i.e., higher toughness).^70,71^

Among the various -ene moieties, the ring strained norbornene has emerged as the most promising one for thiol–ene chemistry. In particular, kinetic and computational analyses have demonstrated that the norbornene group has the highest reaction rate among other common -ene groups such as vinyl ethers, allyl ethers, (meth)acrylates or maleimides.^72−74^ Recently introduced in tomographic printing by Rizzo and colleagues,^70^ thiol-norbornene chemistry significantly improves the printing process performance, making it possible to reduce printing time, light exposure, radical production and guaranteeing better mechanical performances of the printed parts.

Dimerization of hydroxyphenyl groups such as tyrosine,^75−77^ and tyramine^78^ via photooxidation represents another interesting step-growth photo-cross-linking approach, particularly for biological applications. The formation of a covalent bond between two tyrosine residues occurs in native tissue in response to oxidative stress and free radicals, and is therefore increasingly used as a marker of aging and oxidation-related pathologies.^79−81^ In the context of light-based bioprinting, the photoexcitation of a Norrish Type II initiating system (see next Section, 3.3) leads to the abstraction of the hydrogen from the hydroxyl group of the tyrosine phenyl ring, thus forming a reactive tyrosyl radical. The oxygen-centered radical then delocalizes to a carbon-centered radical (two mesomeric forms^81^), and a radical–radical recombination finally leads to the formation of a stable dityrosine cross-link.^82^ Interestingly, the condensation of the aromatic rings can lead to the formation of dimers, trimers, or tetramers depending on the polypeptide structure and length.^83^ In recent years, this process has been used to photo-cross-link unmodified (pristine) protein-based resins featuring a high tyrosine content such as silk or specific decellularized matrices (dECM).^75,76^ Although not yet explored in details in terms of cells and tissue response, and limited to tyrosine-rich polymers, this approach allows the use of pristine protein-based photoresins and native functional groups for cross-linking, thus eliminating the need for chemical functionalization of polymers.

As discussed more in detail in Section 5, it is clear that the whole 3D printing field, from purely material to biological applications, will benefit from a broader use of thiol-norbornene photochemistry and in general step-growth reactions. Although the current trend seems to follow this assumption, the chain-growth photoresins remain popular due to their relatively cheap availability and the requirement of a single component (vinyl-monomer or vinyl-modified polymer) while step-growth systems need the combination of two complementary elements (i.e., thiol and -ene modified polymers). In light of their pros and cons and the specific needs of certain photoresins, various chain-growth and step-growth photoresins have been chosen for a variety of applications, spanning from DVP of metal and ceramics to the use of synthetic and natural polymers for biological applications (discussed in Section 5).

### 3.3 Photoinitiators for DVP

**Figure 7:**
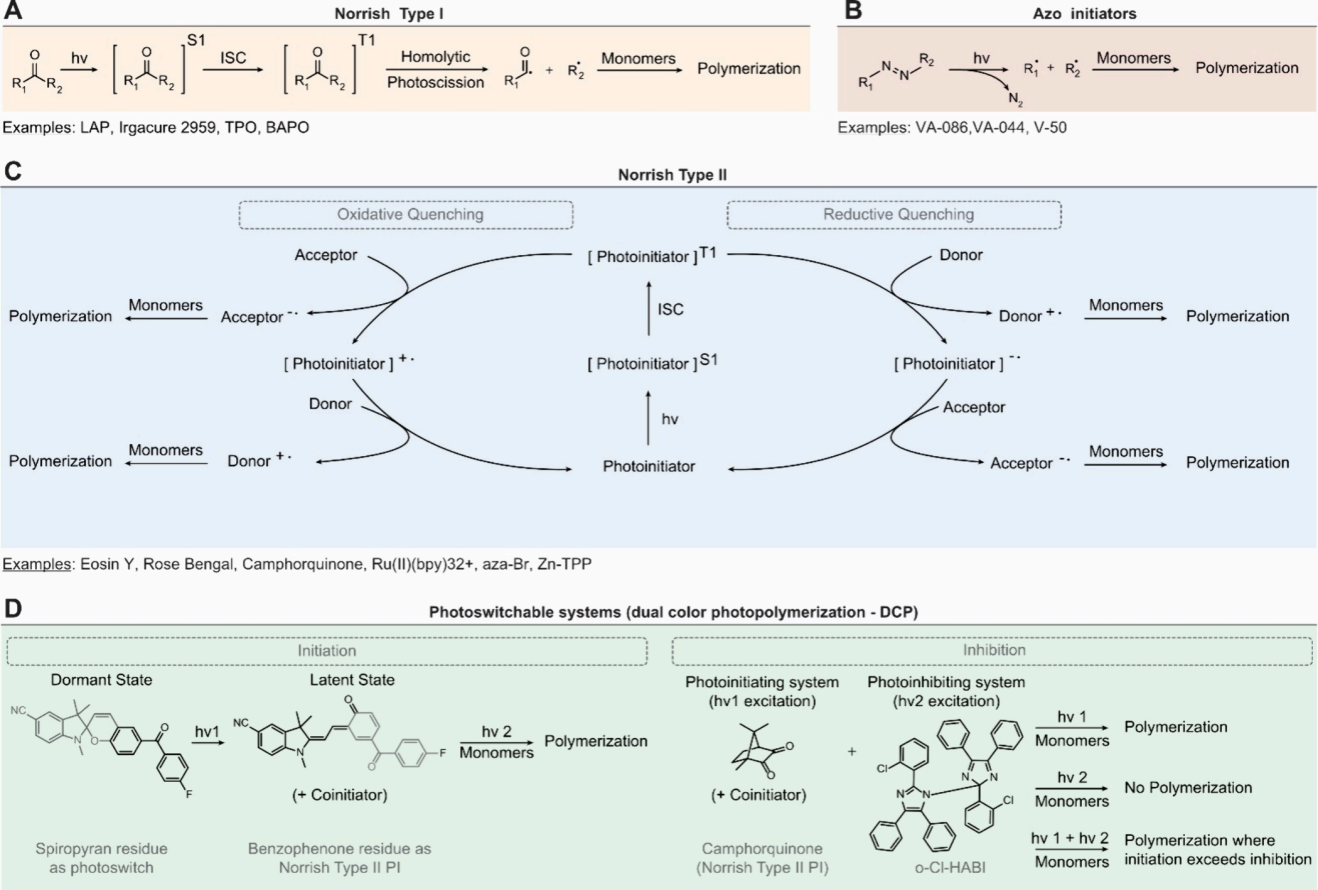
Photoinitiating mechanisms. A. Upon light absorption, the carbonyl group of a Norrish type I photoinitiator is excited to a singlet excited state (S_1_), followed by intersystem crossing (ISC) leading to the triplet excited state (T_1_). The following homolytic photoscission of the α-carbon generates two radical initiating species that trigger the chain-growth or step-growth cross-linking mechanisms. B. Azo initiators are characterized by the azo group (R-N=N-R’) which, upon light exposure, undergoes photofragmentation liberating a nitrogen molecule (N_2_) and leaving two carbon radicals as initiating species. C. Upon light absorption, the Norrish type-II is excited to a triplet state via an ISC process. The following electron transfer processes with co-initiators (photoredox catalysis shown) leads to the formation of the initiating radical species. D. Photoswitchable systems based on the use of two wavelengths to trigger initiation or inhibition mechanisms. The initiation process relies on the excitation of a spiropyran residue (first wavelength, hv1) and of a subsequent type II initiation (second wavelength, hv2) via excitation of the benzophenone residue in the presence of a co-initiator. The inhibition process relies on a champorquinone Type-II photoinitiator (excited with a first wavelength hv1) and an inhibitor (bis[2-(*o*-chlorophenyl)-4,5-diphenylimidazole] (o-Cl-HABI, excited with a second wavelength hv2) that generates lophyl radicals recombining with carbon-center radicals, and thus inhibiting free-radical cross-linking.

**Figure 8:**
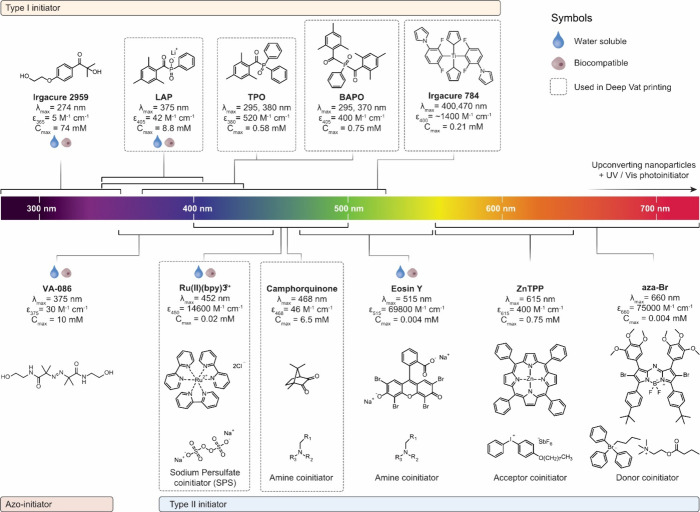
Structure and specifications of various photoinitiators. Abbreviations are LAP: Lithium-Phenyl-2,4,6-trimethylbenzoylphosphinat, BAPO: Phenyl-bis(2,4,6-trimethylbenzoyl)-phosphine oxide, TPO: Diphenyl(2,4,6-trimethylbenzoyl)phosphinoxide, [Ru(II)(bpy)3]^2+^: Tris(bipyridine)ruthenium(II) chloride (used typically with a co-initiator sodium persulfate (SPS)), ZnTPP: Zinc Tetraphenylporphyrin, and aza-Br: A derivative of boron-dipyrromethene cores (BODIPY). VA-086, Irgacure 784, Irgacure 2959 and Eosin Y are commercial names. Each photoinitiator is reported with its typical working window in terms of the wavelength, and its molar extinction coefficient, ε, at the maximum absorption wavelength (λ_max_). Dashed gray boxes represent photoinitiators that have already been used in DVP. The molar extinction coefficient at λ_max_ was used to estimate the photoinitiator theoretically maximum concentration (*C*_max_) that can be used for a tomographic printing process with a path length of 1 mm (eq 2). Notably, as conjugated aromatic compounds, most of the photoinitiators herein displayed have a high absorption in the UVB-C region (<320 nm). Importantly, for some photoinitiators such as TPO, BAPO, and camphorquinone, water-soluble derivatives based on lithium or sodium salts, or addition of a carboxyl group exist and can be used for aqueous photoresins.^2,84,85^

Common photoinitiation mechanisms for the various photoresins used in DVP processes are illustrated in Figure 7. In addition, the structure of the photoinitiating systems, their absorption coefficient at absorption maxima (wavelength) has been illustrated in Figure 8. Although photoinitiators in a photoresin formulation represent a minuscule part (usually used <0.1% w/v), their photochemical properties are crucial for the overall performance of the printing process. An obvious property of the chosen photoinitiators is to have an absorption spectrum that overlaps with the irradiation wavelength. However, since DVP relies on light penetrating deep in the photoresin, a high molar extinction coefficient (ε) of the photoinitiator at the irradiating wavelength can be detrimental. Following Lambert–Beer law on light attenuation (eq 2), a photoresin can be made more transparent by reducing the concentration of the absorbing molecules in the system. It follows that a highly efficient photoinitiator that can be used at low concentration is critical for the rapid cross-linking of a photoresin in DVP. The initiating species formed upon light absorption, radicals or ions, need to be produced to a sufficient extent to form a polymer network in a short period of time, thus limiting their diffusion-induced blurring and extensive light exposure.

#### 3.3.1 Norrish Type-I and Type-II Initiators

Photoinitiators can generally be classified as Norrish Type-I or Norrish Type-II depending on the mechanism generating radical species. For Type-I photoinitiators, upon light absorption the excitation of a carbonyl group leads to a homolytic photochemical cleavage at the alpha-position (α-scission) with the formation of two free radical fragments.^2,86^ Type-II photoinitiating systems are bi- or tricomponent systems that involve hydrogen abstraction or electron transfer with auxiliary molecules (hydrogen donor or co-initiators in the form of electron acceptors/donors), and due to such processes that rely on effective molecule collisions between initiators in the excited state and co-initiators, Type-II based systems are significantly slower than Type-I based systems. To date, most of the work on DVP has been done with Type-I LAP, TPO (Diphenyl(2,4,6-trimethylbenzoyl)phosphinoxide) and Irgacure initiators. In particular, LAP has become the new gold-standard, especially for bioprinting, because of its excellent water solubility (up to ∼5% w/v), biocompatibility and a high molar absorption coefficient in the UV–vis (360–405 nm) range.^2^ Besides commercial Type-I photoinitiators, the field has seen a growing interest in the use of photoredox catalysts with electron donor/acceptor co-initiators. For example, in recent years, a type II bimolecular photoinitiator system based on Ruthenium and co-initiator SPS or ammonium peroxodisulfate (APS) has been exploited because of its absorption in the visible range (400–500 nm, λ_max_ = 452 nm).^87,88^ Upon light absorption, the ruthenium complex Ru(II)(bpy)_3_^2+^ is photolyzed resulting in the formation of Ru(III) and a sulfate radical. Ru(III) is a potent oxidant and has been exploited to dimerize tyrosine residues, thus resulting in cross-linking of tyrosine-rich proteins such as silk.^76,77^ Similarly, tyramine modified polymers can be cross-linked via ruthenium-based photomediated oxidation.^89^ Sulfate radicals on the other hand can directly trigger common chain-growth or step-growth cross-linking chemistries.^90,91^

Importantly, while the field of light-based materials cross-linking has struggled to develop efficient red-shifted Type-I photoinitiators, photoredox catalysts based on xanthene dyes (i.e., Eosin Y or Rose Bengal), boron-dipyrromethene cores (BODIPY) (i.e., aza-Br),^92^ cyanine core (I.e., H-Nu 815), and metal complexes (i.e., Zinc Tetraphenylporphyrin (Zn-TPP))^93^ have emerged as attractive alternatives to produce initiating radical species with long wavelengths and in some cases low light doses. Future work on improving the efficiency of intersystem-crossing (ISC) (i.e., via halogenation), excited state lifetime and water solubility will further strengthen the potential of these photoredox catalyst-based systems and make them usable for DVP. Importantly, as indicated above, such systems rely on electron transfer processes with electron donor and acceptor molecules often used at much higher concentrations than the photoinitiator, thus making the formulations more complex and potentially more toxic than Type-I systems.

#### 3.3.2 Azo Initiators

Azo initiators, similarly to the homolytic dissociation of Norrish Type-I photoinitiators, are another class of molecules that undergo photofragmentation to originate two free radicals. In particular, upon light absorption the photodissociation takes place at the C–N bonds with the release of a nitrogen molecule and alkyl radicals. Although several water-soluble azo compounds are commercially available, their use in 3D printing remains limited, with no report to date for DVP. It is also important to note that, depending on pressure, temperature and amount of azo compound, the nitrogen release can result in the formation of bubbles, thus causing undesired defects in high-resolution printing.

#### 3.3.3 Photoswitchable Photoinitiators

Photoinitiating systems can incorporate one or two molecules that, upon absorption of different wavelengths, can be switched toward initiation or inhibition of photoresin cross-linking. Generally, one component is activated by a first wavelength, and a second wavelength is used to either inhibit or initiate the photo-cross-linking. Dual-wavelength (or dual-color) systems using photoinitiation-photoinhibition mechanisms have been applied to lithographic and DLP-like systems.^10,94,95^ As discussed in previous sections, Regehly and colleagues leveraged a dual-color initiation mechanism to develop a novel DVP method named xolography.^18^ In particular, such dual-color photoinitiator (DCPI) was obtained by integrating a benzophenone initiator core into a spiropyran photoswitch. A first wavelength determines the photoswitching from a dormant (spiropyran) state to a latent (merocyanine) state. The DCPI in latent state can be irradiated with a second wavelength to excite the benzophenone moiety. As the latent state has a defined half-life before switching back to the dormant state via thermal relaxation (*t*_1/2_ = 6 s for DCPI at RT), the second excitation needs to take place right after the first one. The thermal backreaction is necessary for dual-color printing to confine the photo-cross-linking only to those regions where the two irradiations occur in rapid succession, and to otherwise restore the initial, ground conditions. Finally, as described for Norrish Type-II photoinitiators, the excited benzophenone moiety interacts with a co-initiator/auxiliary molecule to form initiating radical species, thus triggering the chain-growth/step-growth cross-linking reactions. Dual color mechanisms appear as a promising research direction for light-based printing, adding an extra level of spatiotemporal control over photo-cross-linking reactions. However, to date the use of these chemistries has not been explored in bioprinting (printing in the presence of cells), possibly due to cytotoxicity of the photoinitiaing components. Future work to investigate the toxicity of these initiating systems and the development of biocompatible ones will significantly expand their potential applications.

With regard to the biocompatibility of photoinitiators, it is known that the radical species generated during the photo-cross-linking process also determine the formation of ROS in aqueous buffers, and can have detrimental effects on cells affecting their DNA, proteins and lipids. Importantly, it has become increasingly clear that near-UV and Vis light (>365 nm) irradiation alone (without initiators) does not affect cells if used within a low light-dose window (intensity <20 mW cm^–2^, exposure seconds to minutes).^57,58^ These observations imply that near-UV and Vis light radical-free chemistries can be exploited for light-based bioprinting processes without deleterious effects on cell fate. For example, Rizzo et al. recently introduced a photoinitiator- and radical-free process based on thiol-Michael addition activated via photouncaging of thiol residues showing excellent cell viability and absence of ROS-associated gene upregulation.^57^ Other radical-free chemistries, ranging from photoinduced hydrazone,^96^ to dimerizations of chromophores^97,98^ and cationic processes^99,100^ have also been explored for light-based cross-linking. However, to date none of these approaches have found their way to Deep Vat bioprinting. Among the various possible reasons, the often-complicated chemical synthesis of the photoresin components as well as their high absorption appear as major limiting factors. The development of less absorbing photosensitive moieties (longer light penetration depth) or alternative radical-free strategies could open new avenues for Deep Vat bioprinting. An alternative route to limit the detrimental effect of radicals on cells is based on macromolecular,^101^ or polymeric initiators.^102^ Binding the initiating species to a polymeric backbone hinders its migration to the cell cytosol, thus drastically reducing photodamage.^103−106^

Recently, although not yet applied to DVP methods, the 3D printing community has shown a growing interest in the use of sustainable natural dyes to replace synthetic photoinitiators. While on one hand this approach aims to reduce the usage of fossil resources and chemicals, biobased molecules could also offer enhanced biocompatibility for various applications, from food packaging to biomedical implants.^107^ In addition, the use of nanoassemblies and nanoparticles as photoinitiator systems, such as upconverting nanoparticles,^108−110^ nanocapsules,^111^ quantum dots,^112^ and carbon dots,^113^ represent an attractive research direction to further expand the palette of visible and red-shifted PIs.

As displayed in Figure 8, all the literature on DVP reported so far have been limited to photoinitiators absorbing in the 360–500 nm range. Considering the benefits of using lower scattering (higher penetration depth) red-shifted wavelengths, it appears clear that DVP (specifically tomographic printing) would significantly benefit from the development of efficient, red-shifted systems. In fact, while on one hand this would potentially lead to the generation of much larger constructs, it would also make it possible to increase the concentration of particles for composite materials, or of cells for bioprinting of high cell density tissues such as liver, kidney or brain. As reported in Figure 8, the current choice of red-shifted photoinitiator is limited to water-insoluble tricomponent systems featuring high-absorbing photoredox catalysts. DVP requires high transparency at the excitation wavelength, and a low concentration of photoinitiators is needed at the expense of the initiation and gelation efficiency. Interestingly, Barner-Kowollik and co-workers have reported on the existence of a mismatch between the absorption profile of a chromophore and its photochemical reactivity, with better performances found to be at red-shifted irradiation compared to their absorbance spectrum.^114^ Therefore, it is theoretically plausible that exciting high absorption photoinitiators away from their absorption maximum (thus leveraging low extinction molar coefficient for DVP) could have minimum or even positive effect on their initiation efficiency. We foresee that future studies in this field would potentially benefit from investigating the so-called action plot analysis (photochemical reactivity resolved by wavelength-by-wavelength mapping), thus potentially opening up possibilities for new types of visible, red-shifted printing with novel or already existing initiators.

## 4 Optimization of the Prints

Like every fabrication technique, DVP has limitations, which mainly stem from either minimizing light attenuation through the volume of the resin vat, preventing light scattering, providing structural support while printing or monitoring the prints. To overcome these limitations, various strategies have been developed, leveraging resin, hardware or software optimizations to improve the printing performance.

### 4.1 Considerations for the Light Source

The first and most important consideration in light-based printing is the choice of the light source which allows the fastest and highest resolution prints. The optical printing resolution is defined by the voxel size (3D pixel) achievable within the photoresin build volume. Owing to diffraction, the size of a projected pixel diverges and increases away from its focal plane, which results in an overlap of the printing pixels in the build volume edges and consequently in a poorer printing resolution. To overcome this limitation and to ensure an isotropic optical printing resolution over the build volume, collimated laser light sources are more suitable for Deep Vat approaches,^18,33,44^ albeit with some exceptions using light emitting diodes (LEDs).^115,116^ In other words, the depth of focus of the projected light patterns is longer with low-etendue (i.e., negligible increase in light cross-section with distance) light sources, such as laser diodes. Laser beams also pack higher energy density and lower diffusion than the light produced by LEDs, and therefore, can also result in faster prints.

The partially coherent LED light or coherent laser beams used within the tomographic printing systems described above exhibit speckle patterns (more pronounced in light-based resins) which are translated to the final projected image sequences. Due to optical self-guidance (eq 11), the light source may cause striation (filamentation) in the tomographically printed objects owing to an optical modulation instability in the photoresins, which can be mitigated by exposing the photoresin build volume to flood illumination at the end of a printing process.^117^ Another way to reduce the striation is through the addition of agents which can increase the refractive index of the resin,^118^ thereby making the change in refractive index upon cross-linking smaller. This way, the optical self-guidance of light is negligible, leading to striation-free constructs. Notably, addition of these refractive index matching agents can also reduce the stiffness of the resins by interfering with the photo-cross-linking processes,^49,118^ and as such, post processing may be needed to improve the stiffness.^49^

### 4.2 Considerations for the Photoresin

A simple optimization to yield higher resolution is reducing the free radical diffusion, and the choice of the resin plays a major role in this. Diffusion of free radical species is directly proportional to the duration of printing (eq 8), and the resins exhibiting faster photo-cross-linkability (e.g., photoclick resins) will result in a better resolution of the printed constructs.^70^ Furthermore, diffusion coefficients are inversely proportional to the resin viscosity (eq 9), and therefore using a higher viscosity resin may yield a better print resolution.^14,41^

**Figure 9:**
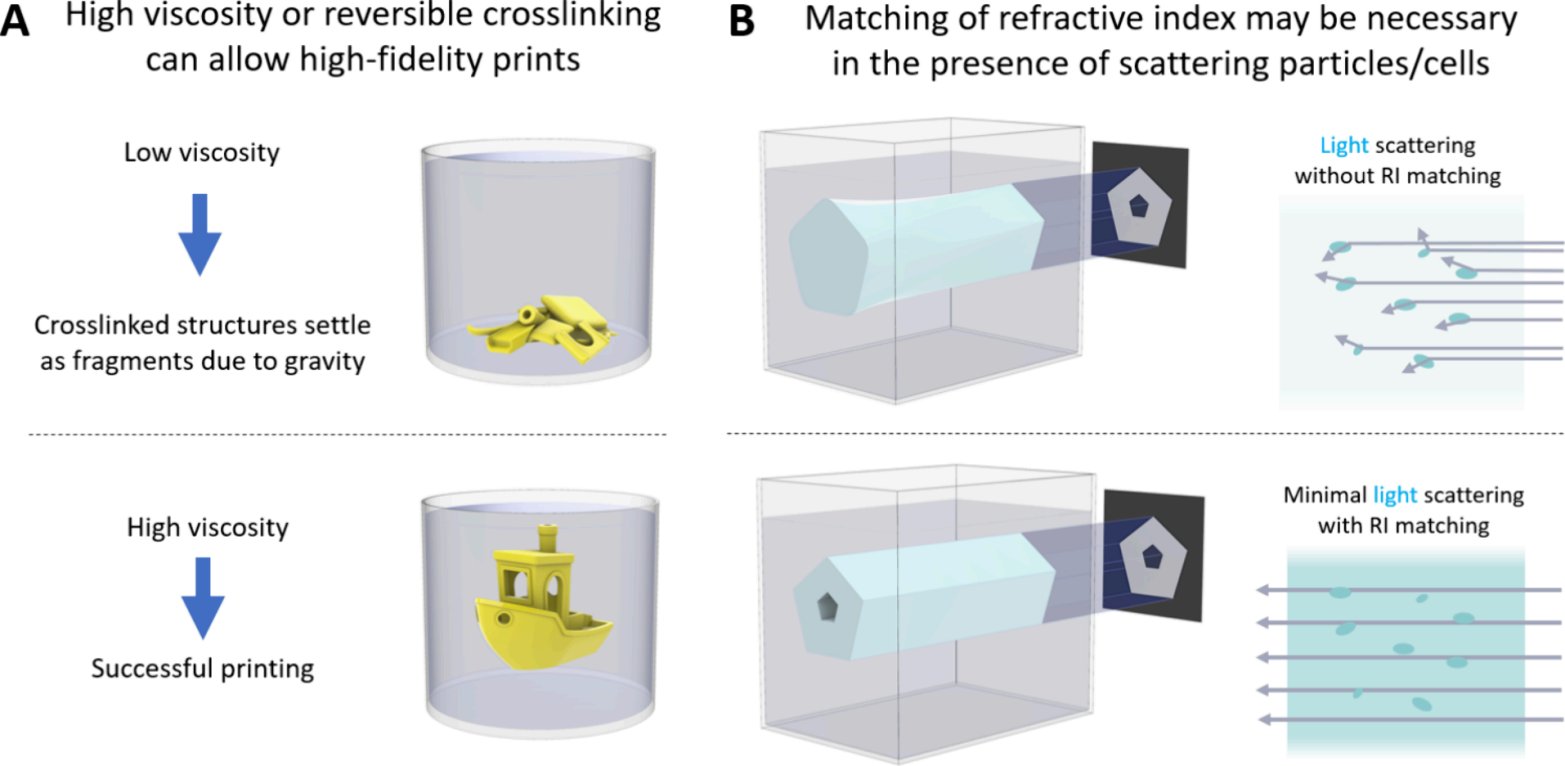
Resin considerations for high resolution DVP. A. High resin viscosity or thermal stabilization (e.g., through the addition of gelatin^120^) prevents the cross-linked structures from settling-down due to gravity, thereby enabling high-fidelity prints. B. Matching of the refractive index of the resin to that of scattering particles such as cells can reduce the scattering of the light transmitted through the resin vial, enabling high-resolution prints.

During the fabrication process, the locally cross-linked polymer becomes denser than the surrounding liquid photoresin, which, in case of a low viscosity resin, may result in sedimentation of the part being generated and a consequent distortion of its shape.^41,119^ The sedimentation speed *v* is inversely proportional to the dynamic viscosity η of the liquid *v* ∝ 1/η. By formulating viscous or shear-thinning resins, the sedimentation time scale becomes longer than the 3D printing time scale,^33^ which prevents part sinking and distortion. Therefore, high viscosity resins^33,119^ or resin compositions which demonstrate thermo-reversible gelation (usually executed by adding sacrificial gelatin^120−123^ or using gelatin-based resin^70,118^) are necessary in tomographic or light sheet-based approaches to prevent sedimentation (illustrated in Figure 9A). For instance, high viscosity acrylate resin formulations such as the dipentaerythritol pentaacrylate or pentaerythritol tetraacrylate have been used for tomographic printing^33^ or xolography,^18^ respectively, to enable high resolution printing. Notably, this requirement of thermo-reversible gelation or high viscosity has not been found to be crucial in FLight, as the cross-linked constructs tend to stick to the wall of the resin vat, which can act as a supporting anchor to the further cross-linked photoresin through the self-focusing of the light.^20,44^ Furthermore, the self-guidance is very fast (usually within seconds) such that the resin viscosity plays a negligible effect on the print resolution.^44^

The photoresins used in DVP are often laden with particles or cells which can cause light scattering. Such cell-induced scattering effects can be partially mitigated by adjusting the photoresin formulation to minimize the refractive index mismatch between the bioresin and the intracellular organelles (illustrated in Figure 9B). Notably, the index matching agents (e.g., Iodixanol) can also result in a reduction in the part stiffness after cross-linking and may need post-treatment to restore stiffness.^49,124^

Recently, great emphasis has been placed on how to achieve larger prints. As shown in the equations for light absorption (eqs 3, 4, and 5), a lower photoinitiator concentration allows minimal absorption throughout the vial, thereby enabling printing of larger objects. However, it should be considered that a lower photoinitiator concentration also results in longer printing duration, and as such, photoclickable resins could enable faster printing with low light attenuation.^70,121^

### 4.3 Considerations for the Process Control

A scaling law for DVP approaches is that the optical absorption length should be equal or longer than the propagation distance *L* (m) of light in the vat of photoresin.^41,119^ Hence, by measuring with a spectrophotometer the molar absorptivity of the resin’s photoinitiating and photoblocking compounds at the illumination wavelength, one can define an upper boundary for the concentration *c* of these photoabsorbing compounds:
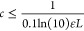
16

However, if the optical absorption length *l*_*a*_ of the resin is shorter than the printing characteristic length, *L*, the center of the photoresin vat will receive significantly less light dose than the edges. To some extent, this can be digitally corrected by boosting the light pattern components that address the center of the resin vat.^35^

Refraction of the projected light patterns by the various interfaces of the optical system, which can be the lens or printing container walls or an immersion bath, may also distort the printed constructs and negatively affect the printing resolution. It is possible to digitally compensate for these distortion effects by ray-tracing the path of the projected lights patterns and digitally resampling them to yield an undistorted 3D light dose distribution into the photoresin build volume.^34,125^

Another consideration is related to the exothermic kinetics of photo-cross-linking. The heat produced could self-accelerate the cross-linking and lead to overcross-linking of the materials, which ultimately affects the resolution. To address this, numerical models have been developed to compute the diffusion (eq 8) and digitally precompensate these autocatalysis effects by optimizing the projected light patterns using deconvolution.^126,127^

Notably, numerical modeling approaches can also be used to complement the considerations for the photoresins when accounting for the part sedimentation or light scattering. For instance, computational models have been developed to predict and compensate for the detrimental effect of part sedimentation in tomographic printing.^128^ A decrease in the light intensity is compensated for in light-sheet stereolithography by graying-out the projection images closer to the light-source.^18^ Light scattering can also be mitigated using digital means, where the scattering properties of cell-laden or composite resins could be calibrated on a small sample, the calibration measurements are then fed to a compensation algorithm that precorrects the light patterns projected during the printing process.^35^ Scattering tends to scramble the high-spatial frequencies, i.e. the fine details, of the projected light patterns, as light propagates deeper in the build volume. The digital compensation effectively boosts these frequencies at different depths to counterbalance the scattering effects. Software optimization of the projected light patterns, prior to the printing process, is also currently being investigated by multiple groups to improve the tomographic printing performance. These optimization approaches include feedback correction and novel algorithms for the computation of the projected light patterns.^129,130^

### 4.4 In Situ Visualization of Printing Progress

**Figure 10:**
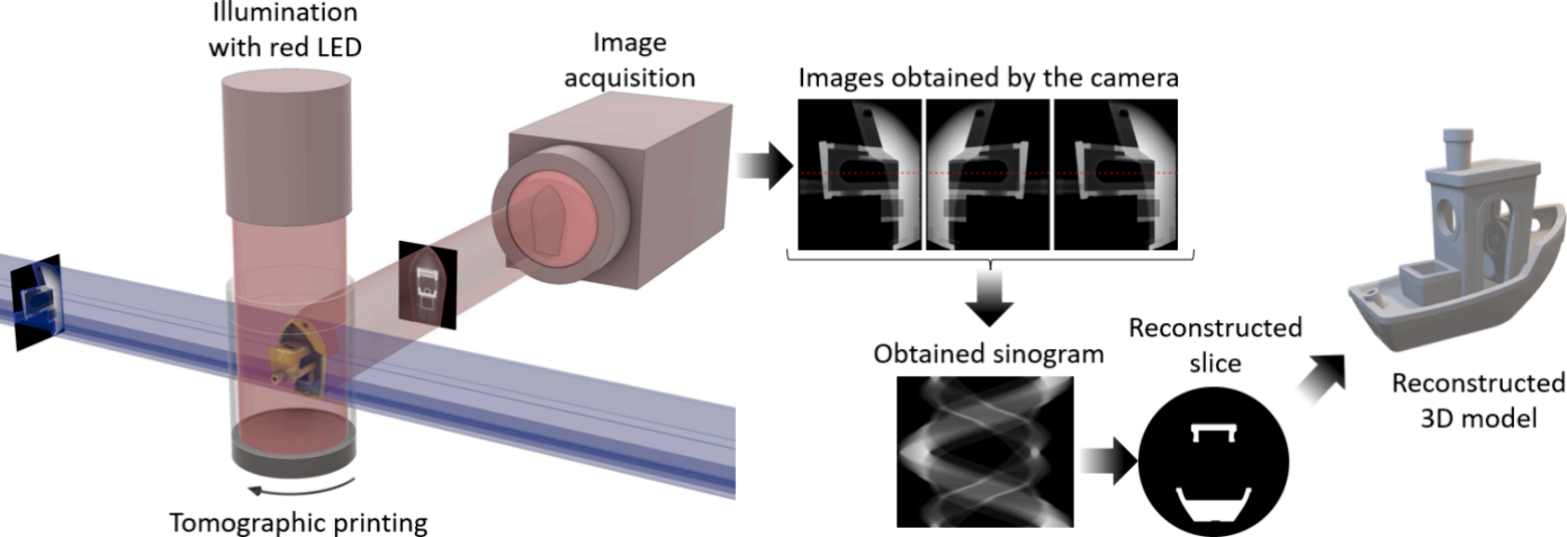
Experimental setup for Optical Scattering Tomography imaging in tomographic printing. The entire vat is illuminated with a red light, and the side-scattered light is directly imaged from the print volume during rotation. The resulting continuous buildup of the object’s sinogram enables subsequent tomographic reconstruction of the printed object.^139^ The model 3D Benchy by Creative-Tools.com is licensed under a Creative Commons Attribution-No Derivatives 4.0 International License.

Given the rapid onset of gelation, means for concomitant inspection and metrology of the ongoing printing process remain a major challenge in the field.^131−133^ This is particularly important as overexposure generally leads to loss of detail and thus low printing fidelity, as well as unnecessary light irradiation of cells. While *posthoc* nondestructive inspection can be performed using micro X-ray computed tomography (μCT),^134^ in situ metrology was largely limited to the assessment of local material properties.^135−137^ Several systems for intermittent imaging have been proposed for layer-by-layer printing, but these systems exhibit extended capture windows and thus do not allow for parallel printing and imaging.^137,138^ Even though the capture of entire models is possible using μCT, imaging regularly requires up to an hour.^134^ An initial attempt at real-time imaging was based on estimating the actual print from transmission images.^33^ To overcome these challenges and allow for rapid and simultaneous image capture during ongoing printing processes, optical scattering tomography reconstructs the print volume by imaging side-scattered light during rotation of the vial containing the photoresin (Figure 10). To correct for light not traveling in parallel to the optical axis, an initial resampling step converts the imaged data into a standard Radon transform. Standard Fourier back-projection is then applied to invert the Radon transform. By adding up the obtained sinograms (filtered Radon transforms) of each layer, a 3D reconstruction of the printed model could be obtained.^139^

In other work, Loterie et al.^33^ visualized the progression of photo-cross-linking employing a monochrome transmission imaging system based on a focused shadowgraphy configuration.^140^ Here, local solidification time was determined by imaging the second spatial derivative of the refractive index using an expanded and collimated red laser. Darkening of voxels (volume elements) beyond an empirically determined intensity threshold from any angular projection was indicative of successful solidification. Importantly, this technique does not provide information concerning density or refractive index of the inhomogeneous areas and is rather limited to a binary cross-linking classification.^33^

Schlieren imaging is yet another method suitable for *in situ* imaging of the printing process. The method was established for both the qualitative and quantitative assessment of fluid properties, and as a means to visualize phenomena in transparent media.^141,142^ Previous reports have further demonstrated that 3D maps of refractive index fields can be generated by processing Schlieren images with tomographic reconstruction techniques, which in turn enables the inference of fluid characteristics such as pressure, density, etc.^143−145^ For the purpose of tomographic printing, tomographic reconstruction from color Schlieren images was employed to track localized material conversion by continuous monitoring of 3D refractive index changes.^48^ Such continuous tracking of RI changes allows spatial and temporal tracking of the photo-cross-linking progression. Compared to the aforementioned method using binary thresholding,^33^ a continuous measurement provides a direct quantification of local errors in the degree of conversion. The latter also proves to be important for real-time projection corrections. Another advantage of Schlieren imaging is its high sensitivity regarding the detection of unwanted background conversion within the resin surrounding the printed model.

## 5 Applications of DVP

The applications of DVP have been widespread, ranging from printing of basic prototypes, to ceramic materials and biological constructs. Notably, there are substantially more applications explored in the field of tomographic printing, since it is the most mature of the DVP techniques. While most process principles are common between the techniques and considering that the field of DVP is rapidly evolving, we have attempted to provide an overview of the applications with a balanced perspective on the different DVP techniques. Table 1 provides a broad overview of the different applications classified based on the cross-linking chemistries, which have been discussed in detail in the following subsections.

**Table 1:** Selected Applications Based on the Type of Photoinitiation System^a^

Cross-linking	Method	Application	PI and wavelength	Free-radical inhibitor, RI matching reagent (*optional)	Max. resolution (min. feature)	References based on photoinitiator (nonexhaustive list)
Chain-growth photo-cross-linking	Multidirection projection and tomographic printing	Biological	LAP (405 nm), TPO (365 or 405 nm)	TEMPO, Iodixanol	100 μm	LAP^49,146−148^
						TPO^149^
		Nonbiological	LAP, TPO (365 or 405 nm), CQ-EDAB (440–460 nm), Irgacure 784, BAPOs, Irgacure 907 (405 or 532 nm)	TEMPO, Iodixanol	20 μm	LAP^35,40,150^
						CQ-EDAB^41,42,151^
						TPO;^33,126,152^ Irgacure 907;^153,154^ Irgacure 784^14^
						BAPO^155^
	FLight	Biological	LAP (405 nm)	-	50 μm	LAP^44^
	Light sheet-based VP	Nonbiological	Dual-color photoinitiator (DCPI, 405 nm +585 nm)	-	20 μm	DCPI,^18,21,16,19^
		Biological	LAP (395 nm)	-	9 μm	LAP^17^
Step-Growth Photo-cross-linking	Multidirection projection and tomographic printing	Biological	LAP (405 nm), TPO (365 nm), Ru-SPS (525 nm)	TEMPO	100 μm	LAP;^70,120,121,156,157^ TPO;^149^ Ru-SPS^76^
		Nonbiological	LAP (405 nm)	Iodixanol	100 μm	LAP^153,158,159^
	FLight	Biological	LAP (405 nm)	-	50 μm	LAP^20,44^

a LAP: Lithium-Phenyl-2,4,6-trimethylbenzoylphosphinat, BAPO: Phenyl-bis(2,4,6-trimethylbenzoyl)-phosphine oxide, TPO: Diphenyl(2,4,6-trimethylbenzoyl)phosphinoxide, Ru-SPS: Ruthenium–Sodium Persulfate, CQ-EDAB: Camphorquinone-Ethyl 4-(dimethylamino)benzoate, TEMPO: (2,2,6,6-tetramethylpiperidin-1-yl)oxidanyl, DCPI: Dual-color photoinitiator.

### 5.1 Chain Growth Cross-Linking for Nonbiomedical Applications

**Figure 11:**
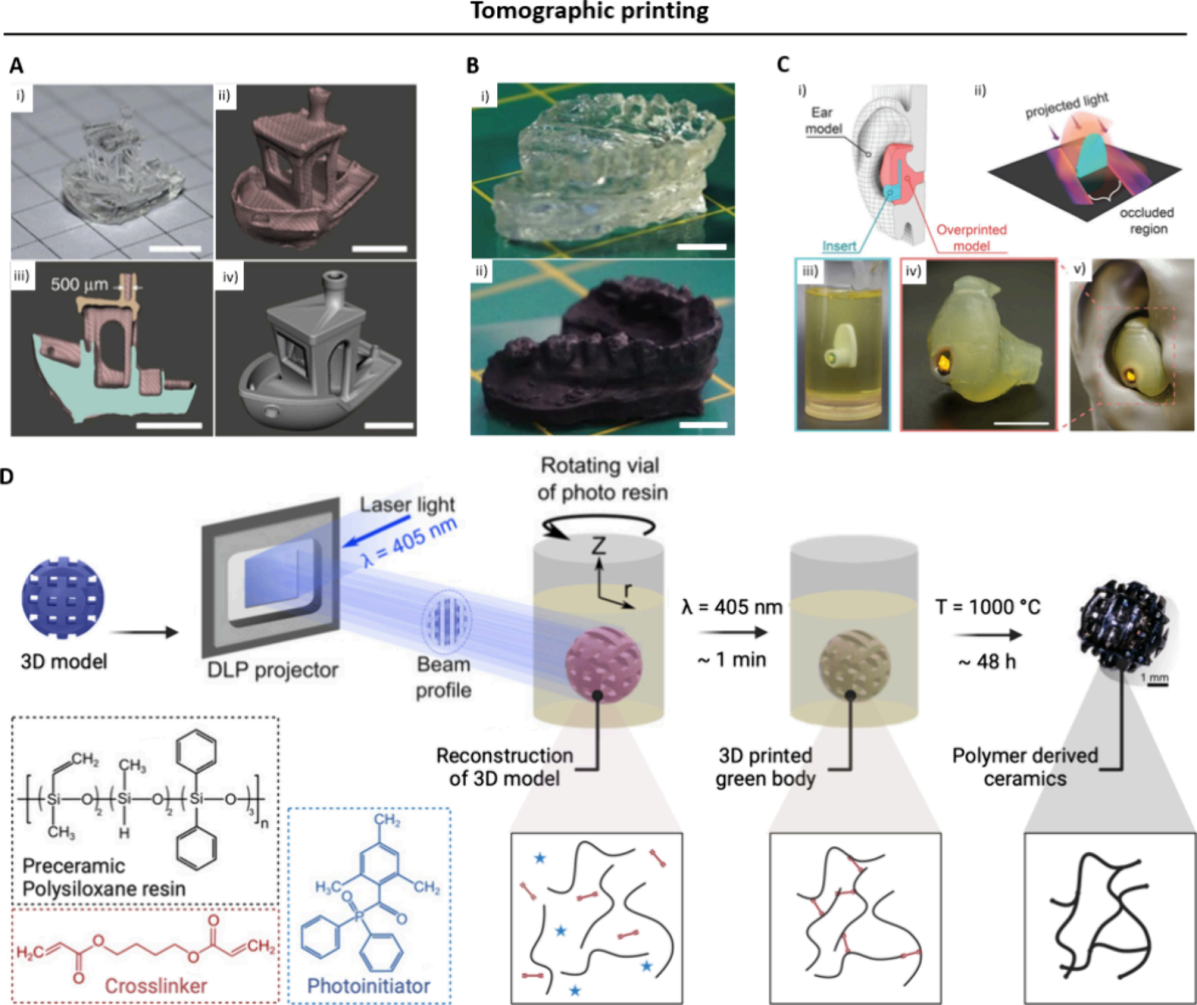
Applications of tomographic printing deploying chain growth photo-cross-linking. A. A 3D Benchy boat model was printed within 25 s in dipentaerythritol pentaacrylate (SR399) acrylic resin (i). Micro-CT rendering (ii) and cross-sectional views (iii) reveal a high printing fidelity compared to the original model (iv). Scale bars: 5 mm^33^ (reproduced from ref^33^ copyright 2020 Nature Portfolio under CC-BY 4.0 [https://creativecommons.org/licenses/by/4.0/]). B. Fabrication of a dental model with complex geometries using tomographic printing and a mixture of two acrylate polymers (bisphenol A glycerolate diacrylate, mixed with poly(ethylene glycol) diacrylate (i). Same model painted for clarity (ii)^41^ (reproduced from ref^41^ copyright 2019 AAAS). C. Tomographic printing is used to overprint the polymer photoresin over an object suspended in the photoresin.^160^ CAD model of the overprinted auditory device (i). The occlusion of projected light by the inserted object is accounted for when computing forward and backprojections (ii). Photograph of suspended insert prior to printing (iii). Overprinted model following curing. Scale bar: 10 mm (iv). Auditory device fitted into the ear canal (v) (reproduced from ref^160^ copyright 2023 Wiley). D. In tomographic printing of silicon oxycarbide ceramics, a 3D model is printed into a rotating vial filled with a photocurable preceramic resin, which consists of polysiloxane, a cross-linker (1,4-butandiol-diacrylate, BDDA), and the photoinitiator diphenyl-(2,4,6-trimethylbenzoyl)-phosphinoxide (TPO). The resulting green body exhibits a stiff polymer network and undergoes further pyrolysis at 1100 °C to yield the polymer derived ceramic. The final pyrolysis step effectively converts the polymeric network of the green body into a silicon oxycarbide ceramic amorphous network. During this step, volatile organic components evaporate (reproduced from ref^165^ copyright 2022 Wiley).

Acrylates were among the first materials employed in DVP applications. They offer several advantages for bioprinting such as facile use, reactivity, and low cost.^160^ By leveraging the high shape fidelity and stiffness of acrylate-based prints, the field has been able to fabricate complex models for biomedical applications. Models of dental retainers or lattice geometries featuring micron-sized details were among the first published examples using tomographic printing (Figure 11A,B).^33,41,152^ Toombs et al. recently demonstrated the fabrication of an auditory device which was suspended in a photoresin and subsequently overprinted to generate a composite object that fit the patient-specific shape of the ear canal (Figure 11C).^160^ To avoid sedimentation of the suspended insert, an ethyl cellulose-based thermoreversible organogel photoresist was developed. Solid ethyl cellulose was dissolved in heated trimethylolpropane triacrylate (TMPTA) and CQ-EDAB (Camphorquinone-Ethyl 4-(dimethylamino)benzoate) photoinitiator. Cooling led to a rearrangement of intermolecular hydrogen bonds of ethyl cellulose, resulting in a porous solid network which trapped the TMPTA monomers. Light exposure then cross-linked the liquid phase and–in a final step–unexposed areas could be removed by heat-induced liquification.^160^ In another application that aimed at the use of tomographic printing for biomedical purposes, Rodríguez-Pombo et al. used the fast cross-linking time of low molecular weight PEGDA (Mn 575 or 700) to fabricate torus- and cylinder-shaped tablets loaded with the drug paracetamol.^161^

Even though plastics and organic materials are easier to shape, they often lack mechanical, chemical, and thermal resistance. For a wide variety of industrial and biomedical applications, ceramics have found widespread use owing to their exceptionally high hardness, as well as thermal and chemical resistance.^162^ More recently, ceramics have also garnered increased interest within the biomedical field due to their high strength and stiffness, in addition to an unrivaled resistance to wear and inertness within living systems, and thermal and chemical resistance.^162−164^.^163,164^ High strength and brittleness of ceramic materials, as well as the high temperatures and pressure required to process them has made the fabrication of complex shapes a long-standing challenge. Historically, such ceramics were manufactured using powder technologies and involved the inclusion of sintering additives, which in turn constrained the range of technical applications.^162^ The introduction of polymer-derived ceramics (PDCs) helped to overcome these manufacturing hurdles. In particular, the methodology is based on the pyrolysis of liquid organosilicon polymers and their subsequent conversion into a PDC, which enables the shaping of preceramic polymers using conventional polymer-forming techniques.^162^ Here, a liquid preceramic polymer precursor was initially photopolymerized into a 3D green body, which corresponds to a low-stiffness version of the final object that is rich in organic components. Following this initial shaping step, the green body was subsequently converted into a PDC through pyrolytic transformation (Figure 11D).^162,165,166^ Pyrolysis of a polymeric precursor requires temperatures around 900–1100 °C.^162^ Several classes of materials have been utilized in the production of PDCs, ranging from binary (SiC, Si_3_N_4_, BN) to ternary (SiCN, SiOC, BCN) and quaternary systems (SiCNO, SiBCN, SiBCO). Silicon oxycarbide (SiOC) has recently been reported for tomographic printing for the purpose of producing complex, centimeter-scale objects. Photo-cross-linking was induced by tomographic back projection. The resin used in the printer was composed of a polysiloxane (SPR 684) with 1,4-butandiol-diacrylate (BDDA) as cross-linker with photoinitiator TPO as the photoinitiator. As previously reported,^166^ pyrolysis-induced mass losses of up to 54% were associated with a significant shrinkage in the PDC volume of up to 31%.^165^ In the case of isotropic shrinkage, however, simple corrections to the 3D model allowed obtaining accurate parts.^167^

Manufacturing of 3D freeform glass objects has become possible by powder-based laser sintering^168^ and molten glass filament deposition techniques.^169^ These techniques are superior in producing complex shapes when compared to traditional glass shaping technologies, such as blowing or casting. However, they still require the glass to be in a molten state at the time of printing. In case of refractive index-matching, silica nanoparticle-loaded precursor photoresins and liquid monomer binders can also be used in tomographic printing. Toombs et al. produced high-resolution fused silica components by tomographic illumination of a photopolymer–silica nanocomposite with subsequent sintering.^151^ Here, tomographic printing of a 3D model is followed by the conversion of the green part into a brown part by debinding at 600 °C, and a final sintering step at 1300 °C which converts the brown part into a dense silica part. This particular system comprised a photocurable microstereolithography silica glass nanocomposite (Glassomer μSL v2.0) that consisted of a liquid monomeric photocurable binder matrix and 35 vol % solid amorphous spherical silica nanoparticles (40 nm diameter). The binder matrix provided support to the nanoparticles in the printed construct, and was composed of trimethylolpropane triacrylate and hydroxyethyl methacrylate with CQ-EDAB photoiniaitor system, as well as the radical scavenger TEMPO.^151^ The addition of TEMPO significantly increased lithographic contrast of the prints and allowed extending the induction time, i.e. the period during which selective material conversion is inhibited due to the presence of molecular oxygen.^14,33,41^ When printing complex cubic lattice structures using a silica glass nanocomposite material, minimal positive feature sizes of 50 and 20 μm were achieved for nanocomposite and monomeric materials, respectively. The improved features sizes in polymeric photoresins in the absence of solid nanoparticles could be attributed to reduced light-scattering, as well as lower resin precursor viscosity and reduced brittleness of the green body.^151^

**Figure 12:**
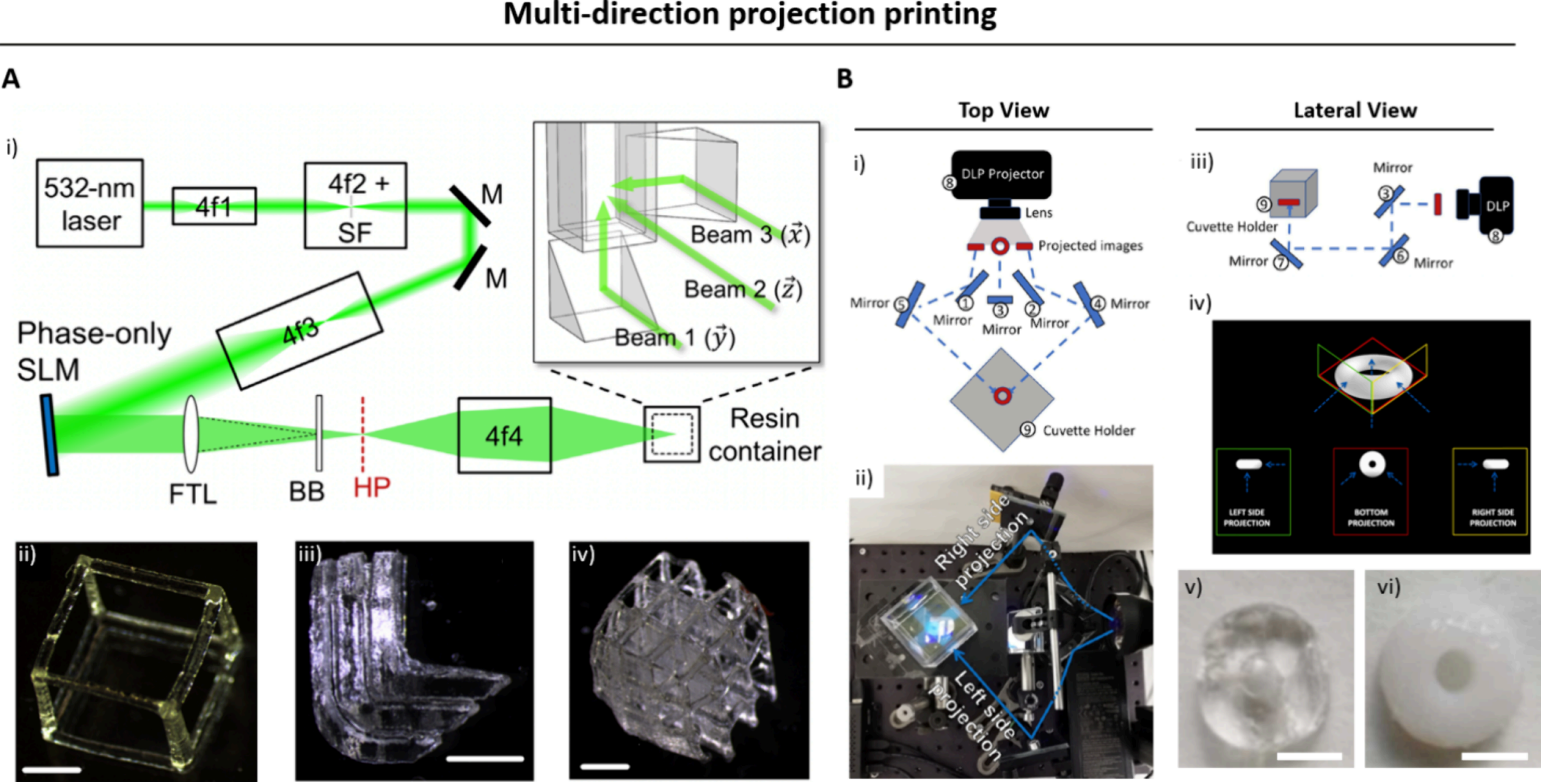
Applications of multidirection projection printing deploying chain growth photo-cross-linking. A. Schematic of the multidirection holographic patterning system using the superposition of three orthogonal beams which intersect in the resin (i). Subregions of a holographic image are deflected by mirrors at an angle of 45° to generate individual beams (i, insert). SLM, spatial light modulator; FTL, Fourier transform lens; BB, beam block that excludes undiffracted light; HP, hologram plane; 4fN, telescope lens pairs (“4-f” configuration); SF, spatial filter (i). Multidirection projection printing was used to fabricate various geometries of increasing complexity (ii-iv, scale bars: 2 mm) (reproduced from ref^14^ copyright 2017 AAAS). B. Design principles of the printer for multidirection projection printing. Top (i,ii) and lateral view (iii, (iv), with blue arrows referring to the optical projection path. (1) and (5) refer to mirrors for the right side projection; (2) and (4) refer to mirrors for the left side projection; (3), (6), and (7) refer to mirrors for the bottom projection. Schematic representing optical fields projected onto the photoresin using three orthogonal light beams (iv). Photographs of printed torus-shaped Printlets with decreasing PEGDA content (Mn = 575; 85% (v), 33% (vi) PEGDA (% w/w)). (reproduced from ref^40^ copyright 2022 Elsevier under CC-BY 4.0).

While tomographic printing has amassed more applications, it is important to note the applications of the multidirection projection technique. Shusteff and colleagues employed this fabrication modality to generate both symmetric as well as asymmetric models, including cubes, cantilevers, and lattices (Figure 12A, panel ii-iv). The printed structures were fabricated from low-viscosity PEGDA (Mn = 250; μ ≈ 12 cP) using Irgacure 784 as photoinitiator and a 532 nm wavelength laser source. This system was used to fabricate geometries with self-supporting positive features in the range of ∼300 to 400 μm. However, the authors speculated that further optimization of the photoresin to balance viscosity and O_2_ diffusivity would enable printing closer to the diffraction limit of the optical system (20 to 50 μm).^14^ The high fabrication speed of multidirection projection printing was recently employed by Rodríguez-Pombo et al.^40,161^ to fabricate drug-loaded torus-shaped tablets (Printlets) based on PEGDA (Mn = 575) formulations of various concentration, using LAP as photoinitiator (Figure 12B). Prior to printing, a custom in-house software was utilized to generate three orthogonal projections from the provided 3D geometry and employed a similar transverse intensity profile correction to the individual light beams to compensate for the limited axial resolution of orthogonal beams when superimposed in the photoresin. Furthermore, the optical system used a 385 nm laser source and an array of UV-reflecting mirrors to divide the projected image into three parts (Figure 12B, i-v). Print times ranged from 7–12 s, and generally decreased for lower PEGDA concentrations (Figure 12B, vi-viii). This rapid and customizable fabrication method, coupled with high-throughput capabilities, was developed to pave the way for new possibilities regarding the decentralized production of personalized medicine.^40^

**Figure 13:**
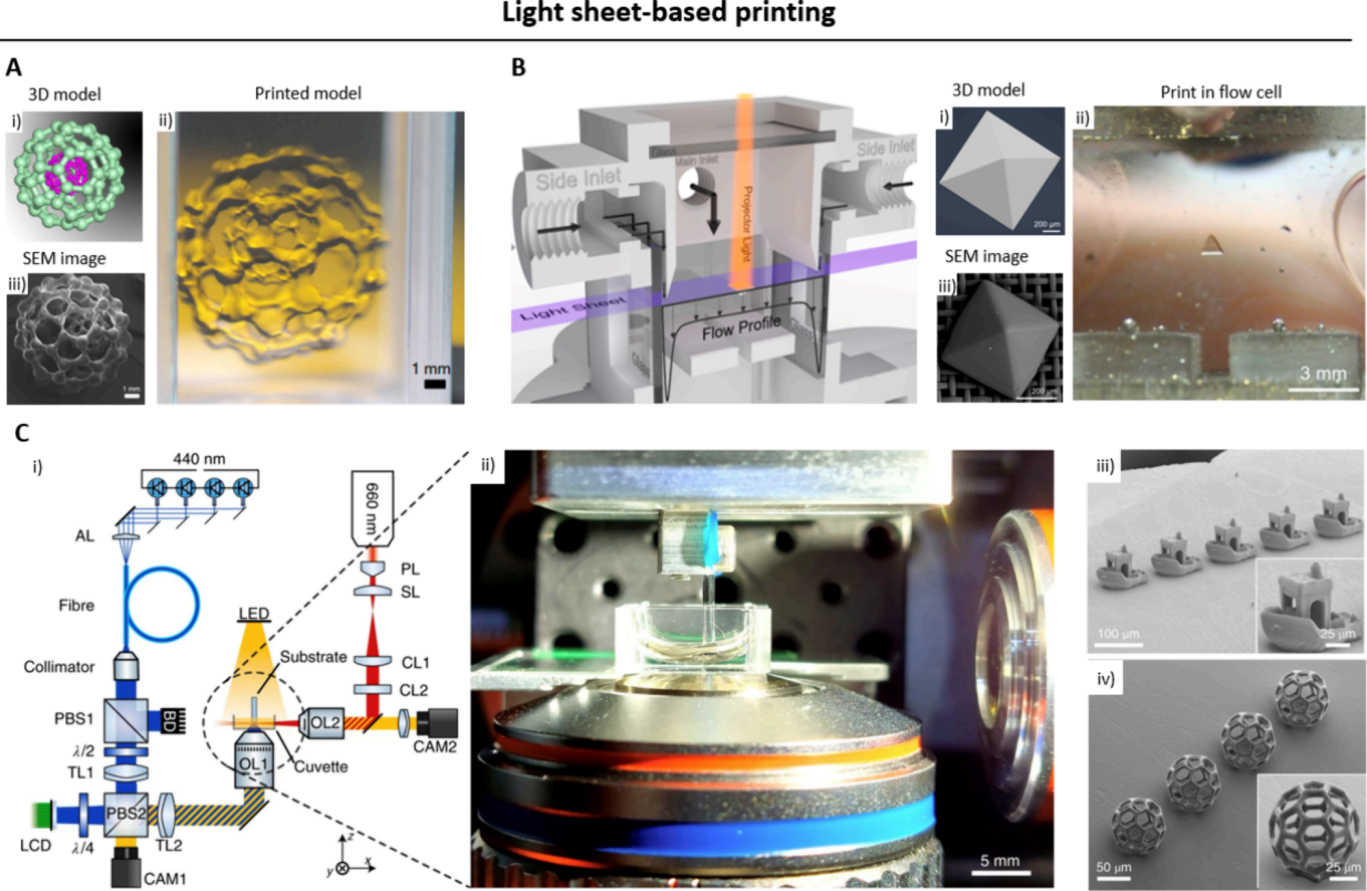
Applications of light sheet printing deploying chain growth photo-cross-linking. A. Application of xolography (dual-wavelength light sheet-based printing): model of nested fullerene (i), printed model before postcuring (ii), and after postcuring as visualized with SEM (iii) (reproduced from ref^18^ Copyright 2020 Springer Nature). B. Application of xolography in flow to enable continuous high-throughput printing: 3D model of the print (i), in situ observations of the print in the flow cell (ii), and SEM image showing high printing fidelity and smooth surfaces (iii) (reproduced from ref^21^ copyright 2023 Wiley under CC-BY 4.0). C. Light-sheet 3D printing setup consisting of two laser beam paths.^54^ The stationary red-light-sheet beam (λ2 = 640 nm) and the blue-light beam (λ1 = 440 nm) projecting slices of the object cross inside a cuboid cuvette chamber (i). The cuvette is mounted on a glass coverslip and positioned between the orthogonal arrangement of objective lenses (OL1, OL2) (ii). Printing of a series of 3D Benchy structures showing overhangs and small negative features (iii). Printing of a series of buckyball structures with diameters of 80 μm (iv) (reproduced from ref^54^ copyright 2022 Springer Nature).

In parallel to the advent of tomographic printing, xolography has been introduced as a light sheet-based technique to generate 3D objects with complex geometries. Xolography is based on photoswitchable photoinitiators to induce local photo-cross-linking upon excitation by intersecting light beams of different wavelengths (c.f. Section 2). In their initial work, Reghely and co-workers employed pentaerythritol tetraacrylate (PETA)- and diurethane dimethacrylate (UDMA)-based resins to print complex structures such as nested fullerenes which exhibited defined structures in multiple spatial directions (Figure 13A).^18^ While impressive in its achievable resolution, this initial demonstration of xolography did not feature cytocompatible applications. Further work advanced the initial setup into a continuous process, where dual-color photo-cross-linking was performed inside a flow cell (FlowXube) with continuously flowing resin (Figure 13B).^21^ The technique enabled an upscaling in the production rate while maintaining high resolution of the printed parts. Most recently, light-sheet bioprinting via two-color two-step absorption has been demonstrated, which is akin to the parallelization of a one-color two step absorption setup utilizing a low-power continuous-wave laser for focus-scanning 3D printing (Figure 13C).^54^ Photo-cross-linking is limited to areas where a continuous-wave light-sheet (660 nm) overlaps with the continuous-wave projection laser (440 nm). To avoid sedimentation, the method benefits from highly viscous photoresins such as dipentaerythritol hexaacrylate (DPEHA) with a viscosity of 6.0 PaS.

### 5.2 Chain Growth Cross-Linking for Biomedical Applications

When the printing process involves biological components such as cells or proteins, and/or the final use of the printed construct is to be implanted into an organism, there are several important considerations to be made in addition to the general photoresin requirements described in Sections 2 and 3. The delicacy and complexity of the biological world pose limitations on the printing conditions, the photochemistry choice as well as the choice of the photoresin components. For example, when printing in the presence of cells, free-radical initiated processes which use extended light exposure result in accumulation of ROS that are known to have cytotoxic effects.^57^ On the other hand, although often described as cytotoxic, light exposure (in the absence of a photoinitiator) in the UV–vis range (365–405 nm) that is often used in bioprinting does not per-se induce significant cell damage when used below 20 mW/cm^2^ of light intensity.^58^

Gelatin methacryloyl (GelMA) is a common, highly biocompatible material used in biomedical applications, and has found widespread application in the field of tissue engineering owing to its low immunogenicity and the presence of cell-adhesive motifs.^170,171^ Its versatility has been proven particularly useful in the biomedical field using extrusion-based and light-based fabrication techniques.^113,122,172,173^ GelMA is routinely synthesized by reacting lysyl side-chains of gelatin with methacrylic anhydride.^174^ The generation of stable hydrogels requires the optical cross-linking of incorporated methacryloyl groups in the presence of a photoinitiator.^170^

**Figure 14:**
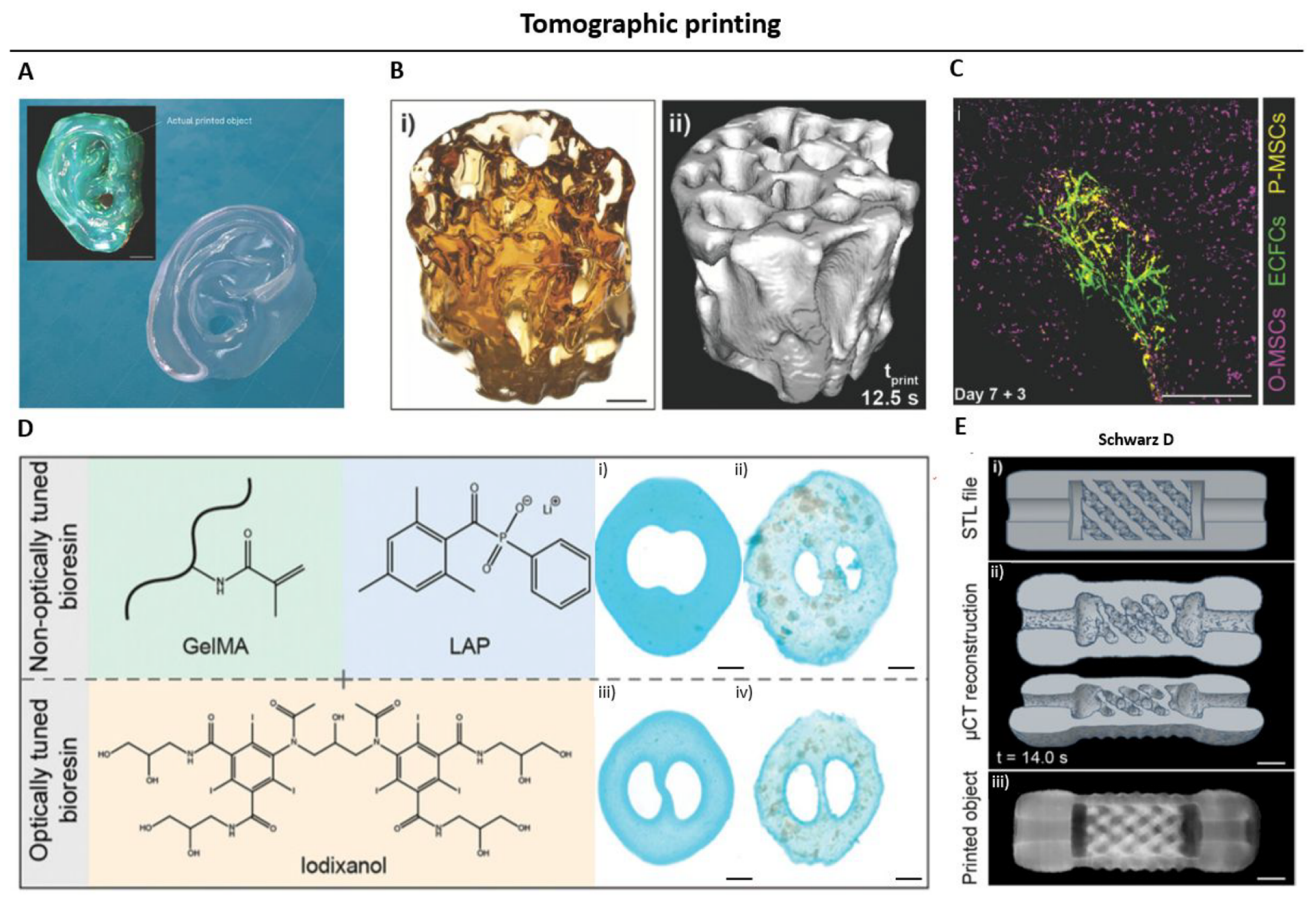
Tomographic printing of chain-growth bioresins. A. Stereomicrograph of printed human auricle model (scale bar: 2 mm).^148^ B. The print of a complex trabecular bone model exhibits high shape fidelity postprinting (i), as visualized using μCT imaging (ii) (scale bar: 2 mm).^148^ C. Trabecular bone models seeded with mesenchymal stromal cells (MSCs) and endothelial cells (ECFCs) supported the formation of capillary structures (scale bar: 1 mm)^148^ (reproduced from ref^148^ copyright 2019 Wiley). D. Comparison of the conventional and modified bioresin containing the index-matching additive iodixanol. Stereomicrographs of nonoptically tuned (i,ii) and optically tuned (iii,iv) bioresins (scale bars: 1 mm).^49^ E. Optical tuning of photoresin facilitated VBP of complex perfusable structures. STL models (i), μCT-based 3D reconstructions (ii), and macro-photographs (iii) Schwarz D architecture (scale bars: 2 mm) (reproduced from ref^49^ copyright 2022 Wiley under CC-BY 4.0).

Bernal et al.^148^ reported the development of a GelMA-based bioresin using LAP. The authors demonstrated fast tomographic printing of centimeter-sized tissue models such as the human auricle (Figure 14A). Moreover, an anatomically shaped, cell-laden trabecular bone model was printed to demonstrate printing fidelity (∼95%), as well as cell viability and the development of angiogenic sprouts within the printed construct (Figure 14B,C). This study further demonstrated functional maturation of GelMA-based resins containing 10 mio cells/mL articular cartilage progenitor cells (ACPCs) into a fibrocartilage-like matrix over a period of 28 days.^49^ Recently, osteogenic differentiation and maturation of perfusable cell-laden constructs into bone-like constructs was also shown.^120,147^ Long-term monitoring over a period of 42 days resulted in upregulation of osteocytic and osteoblastic markers, thus demonstrating the suitability of tomographic printing as a platform for bone tissue-engineering.

A long-standing problem facing extrusion-based printing is shear stress-induced damage to cells when utilizing high-viscosity bioinks, and similarly the fragmentation of fragile organoids.^175,176^ Tomographic printing has been utilized to overcome this hurdle and print complex constructs containing morphologically intact liver organoids while preserving ECM components deposited into the extracellular space prior to printing.^49^ Light scattering due to the presence of organoids initially resulted in off-target cross-linking, and consequently low printing resolution. This scattering effect depends on the length of the scattering mean free path at the chosen wavelength, which is inversely proportional to density and size of cellular components in the resin.^177^ An algorithm-based approach that iteratively corrects the projected patterns (discussed in Section 5) has been developed, but only works for particles with homogeneous size distribution.^35^ Using an index matching compound (Iodixanol) the authors were able to reduce light-scattering effects introduced by organoids of heterogeneous size (Figure 14D). The authors further demonstrated tomographic printing of various gyroidal lattice structures that featured high surface-to-volume ratios and interconnected pores, where modulation of geometrical parameters allowed control over the flow profiles through the porous construct (Figure 14E).

**Figure 15:**
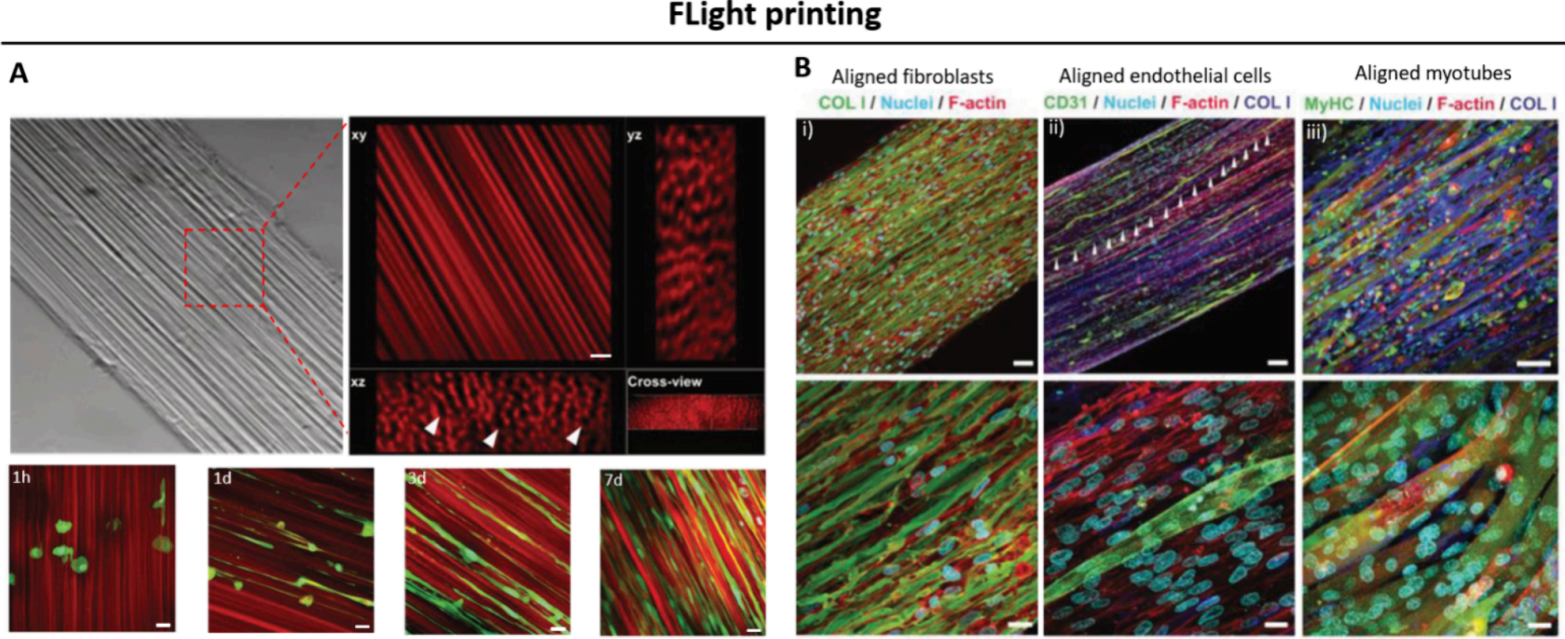
FLight printing of chain-growth bioresins. A. Unidirectional microfilament networks created with FLight using a GelMA photoresin. Microchannels between microfilaments can be washed out after completion of the print (white arrows). NHDFs (green) in cell-laden hydrogels rapidly migrate into and along the microchannels (scale bars: 20 μm). B. Microfilaments provide cell guidance and allow for maturation of highly aligned cellular constructs that incorporate NHDFs (normal human dermal fibroblasts (i), endothelial cells (ii), or myoblasts (iii), respectively. Scale bars upper panel: 100 μm, lower panel: 20 μm. Scale bars upper panel: 100 μm, lower panel: 20 μm. Images reproduced from ref^44^ copyright 2022 Wiley under CC-BY 4.0.

Filamented light (FLight) bioprinting has just recently been introduced as a means for the rapid biofabrication of tissue-engineered constructs composed of highly aligned, unidirectional microfilament networks (Figure 15A).^44^ This fabrication method proved to be highly supportive for rapid cell infiltration into, and migration along the microchannels.^34^ Moreover, depending on the spatial coherence length of the light beam, both diameter of microfilaments and microchannels could be easily tuned between 3–20 μm.^44^ FLight is especially intriguing in the biofabrication of cell-instructive anisotropic tissues (muscle, tendon, articular cartilage, nerves, etc.), which so far were limited in scalability, achievable aspect ratio and alignment of the engineered microstrands.^178−181^Figure 15A shows the FLight-generated constructs based on resins made of GelMA. The constructs feature microfilaments throughout their length, whose diameter (5–30 μm) is close to the size of single cells. The microfilaments provide excellent topographical cues for instructing cell alignment and anisotropic ECM secretion and organization. Within the FLight-fabricated constructs (Figure 15A), the encapsulated cells aligned along the microfilaments and exhibit nuclear deformation and orientation, ECM deposition pertaining to connective tissues, tendons and muscles (Figure 15B). For example, the fibroblast-laden constructs demonstrated high nuclear aspect ratios and collagen I deposition, and myoblast-laden constructs demonstrated aligned multinucleated myotubes (Figure 15B).

**Figure 16:**
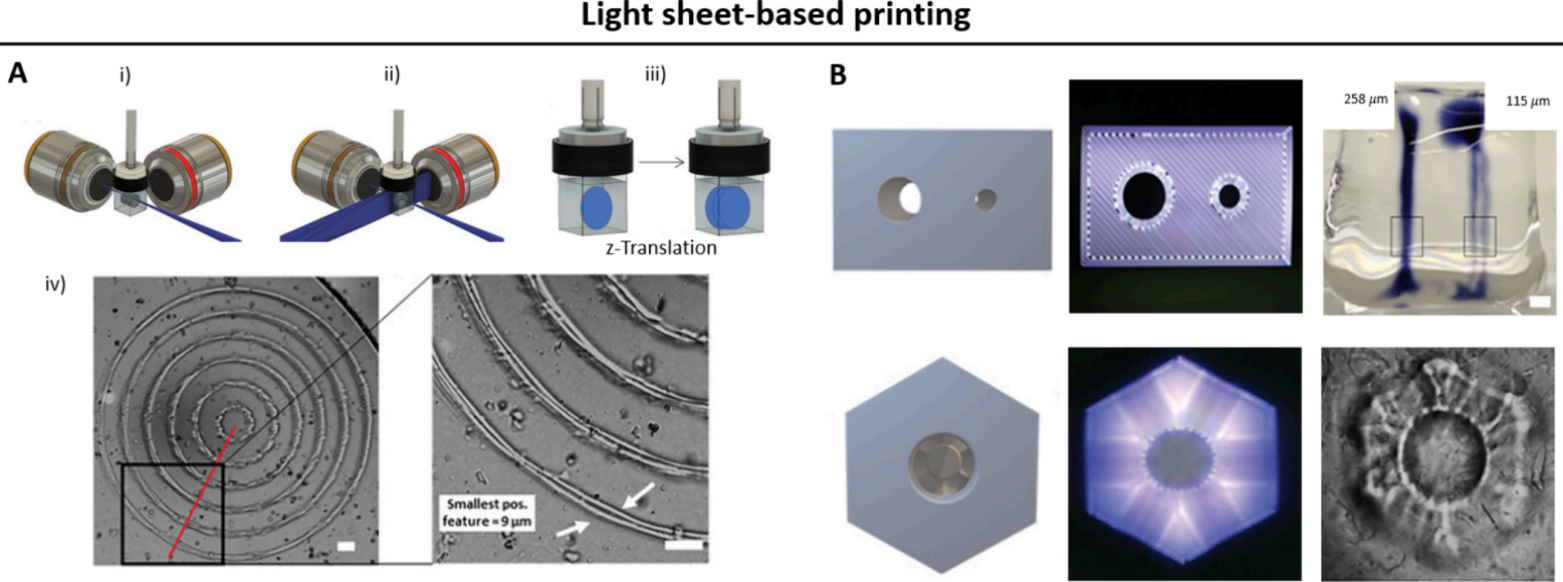
Light sheet-based printing of chain growth bioresins. A. In the light-sheet bioprinter setup, the cuvette containing the bioink is placed at the crossing of the scanned laser beam and the static light sheet (i-ii). Printing proceeds in a layer-by-layer process, involving xy-patterning followed by z-translation (iii). Example printed constructs featuring concentric rings, executed through laser scanning along a glass slide (iv). B. Printing of a liver lobule model with well-defined edges (i). Perfusable construct with nominal channel diameters of 1 mm and 0.5 mm showed partial occlusion following prints and Trypan blue perfusion (ii). Scale bar 1 mm. Images reproduced from ref^17^ copyright 2023 Wiley under CC-BY 4.0.

Light sheet-based printing was also recently leveraged to bioprint cellular constructs. In their work, Hafa et al.^17^ produced full-thickness skin constructs and achieved high cell viability directly postprinting (90%), with only slight decreases in viability for extended culturing periods (83% at d 7). For shallow structures that were printed directly onto glass slides, the system allowed for printing speeds of 0.66 mm^3^/s and resolution of 9 μm (along the plane of the glass slides) (Figure 16A,B). Compared with the two-wavelength system,^54^ the light sheet setup combines a static light sheet (λ = 405 nm) and a scanned light pattern (λ = 395 nm). Whereas the static light sheet selectively exposes single xy-planes with a light dose below the cross-linking threshold, the scanned light sheet sufficiently exposes the photocurable resin to selectively surpass the cross-linking threshold in the overlapping region. Interestingly, no significant difference in cell viability was observed regarding the use of a single scanning beam or the use of both a static light sheet and scanning beam. Owing to the cross-linking wavelengths used, the system is compatible with commonly used photo-cross-linking chemistries. Additional wavelengths allow further flexibility regarding other photoinitiator chemistries.^17^

### 5.3 Step Growth Cross-Linking for Nonbiomedical Applications

**Figure 17:**
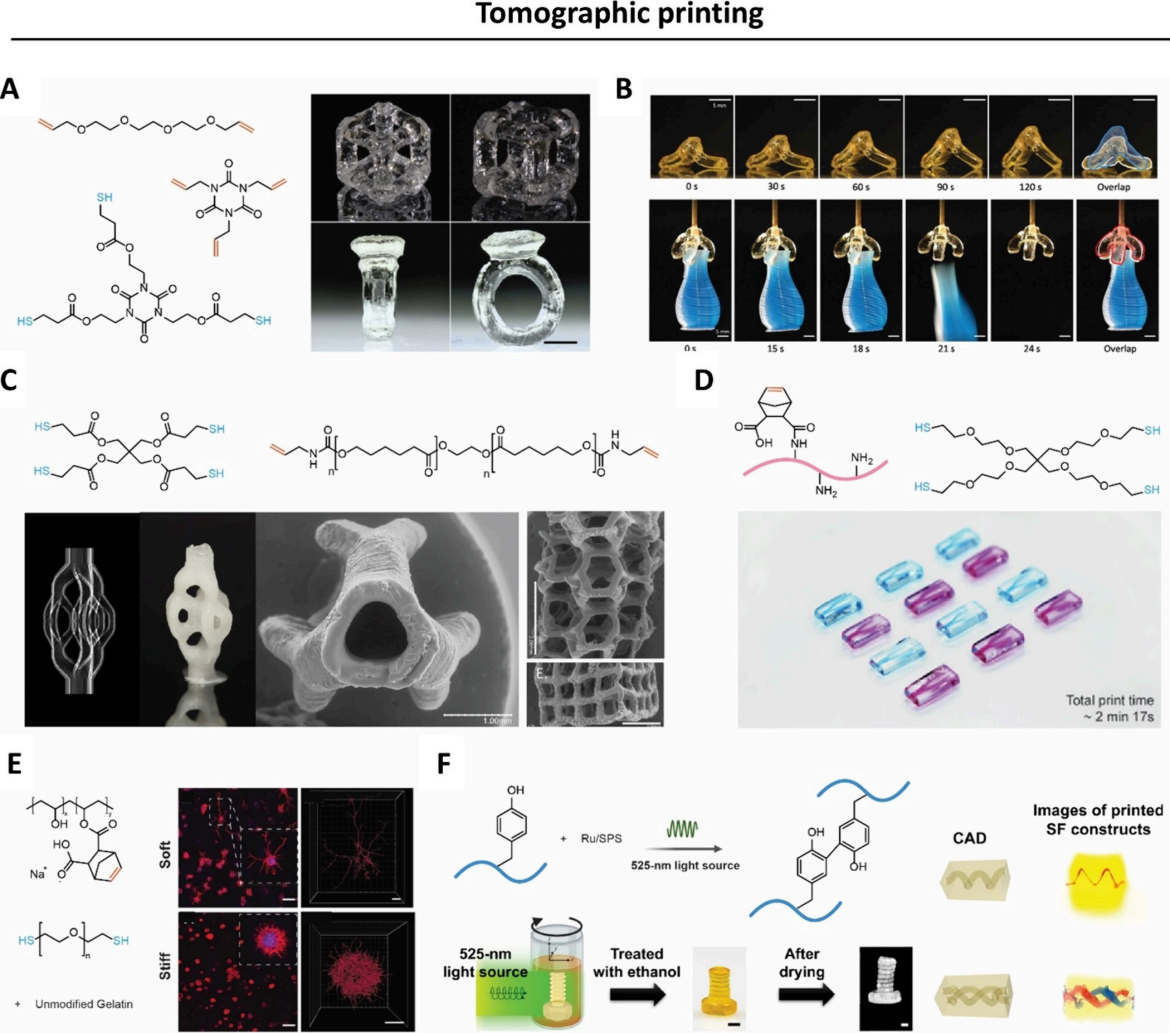
Applications of step-growth photoresins. A. Tomographic printing with thiol-allyl based photoresin. Scale bar: 5 mm (reproduced from ref^153^ Copyright 2020 Wiley). B. Gripper thiol-allyl based shape memory polymers (SMP) showing thermal actuation. Scale bar: 5 mm^158^ (CC BY-NC 3.0). C. Synthetic biocompatible scaffolds composed of allyl-terminated polycaprolactone (PCL) and thiolated cross-linker. Scale bars: 1 mm (middle and bottom right image), 2 mm (top right) (reproduced from ref^149^ Copyright 2023 Wiley). D. Thiol-norbornene based gelatin (simplified pink backbone) photoresin for fast, high throughput printing of perfusable models. (models are 2 cm in length) (reproduced from ref^70^ Copyright 2021 Wiley). E. Thiol-norbornene based PVA photoresin for tomographic printing of soft matrices resulting in improved 3D cell growth. Scale bars: 50 μm, 10 μm (inserts) (reproduced from ref^120^ Copyright 2023 Wiley). **F.** Tomographic printing of pristine silk (simplified blue backbone) via Ru/SPS initiating system and visible light leading to tyrosine dimerization, and postprinting ethanol/drying treatment to obtain stiffer constructs. Scale bars: 500 μm. (reproduced from ref^76^ copyright 2023 Nature Portfolio CC BY-NC 4.0).

As discussed in Section 3, step-growth photoresins offer several advantages when compared to chain-growth ones, thus naturally representing a system of choice also for DVP. For instance, the faster gelation kinetics, the more homogeneous networks showing improved toughness, less shrinkage behavior and narrower glass transition temperatures can be beneficial for a variety of applications, from fabrication of elastomeric constructs to bioprinting. In particular, the use of step-growth photoresins in DVP has so far been limited to the well-established thiol–ene chemistry. Cook et al. introduced the printing of thiol–ene photoresins by means of tomographic printing,^153^ showing improved and highly tunable mechanical properties using allyl and thiol terminated short multifunctional monomers when compared to acrylate counterparts (Figure 17A). Later, the same photoresin formulation was further investigated to generate stimuli (temperature) responsive constructs.^158^ By varying the ratio between di- and trifunctional allyl monomers, and keeping the overall allyl:thiol ratio constant (1:1), the authors were able to tune the glass transition temperature (*T*_*g*_) of the resulting shape memory polymer (SMP) materials from −53 to 55 °C, and finally show thermal actuation of a tomographically printed gripper (Figure 17B). Being intrinsically layer-free, tomographic printing appears as a promising technique to generate defect-free shape memory polymers (SMPs), thus overcoming the issue of anisotropic responses arising from layering defects.

### 5.4 Step Growth Cross-Linking for Biomedical Applications

Step-growth chemistries represent an optimal choice for biomedical applications since their fast kinetics reduces the light exposure time and consequently radical formation. Recently, Thijssen et al. exploited allyl-thiol step-growth cross-linking to tomographically print a photoresin composed of allyl-terminated polycaprolactone (PCL) and a small tetrafunctional thiolated cross-linker (pentaerythritol tetrakis(3-mercaptopropionate))^149^ (Figure 17C). The resulting photo-cross-linked parts showed much improved, better predictable, and tunable mechanical properties compared to their acrylate cross-linked counterparts. Interestingly, with PCL being known and used as a biocompatible and biodegradable component for resorbable synthetic scaffold in biomedical applications, the authors also confirmed such properties for their thiol–ene constructs upon *in vivo* implantation in mice.

Allyl-thiol step-growth cross-linking was recently exploited also by Ciancosi et al. in a photoresin composed of allyl-gelatin (Gel-AGE) and thiolated PEG.^156^ By tailoring the polymer content and the allyl to thiol ratio, the authors reported tomographic printing of exceptionally soft matrices (200–1000 Pa) in tens of seconds (20–50 s). Delicate, differentiated adipocytes were embedded in Gel-AGE based photoresin and printed with high viability (>90%) as proof-of-concept for tomographic bioprinting in the engineering of soft tissues such as brain, lung, breast and endothelial tissues. Although allyl functionalities proved to be applicable for thiol–ene based cross-linking, the use of strain-promoted -enes such as norbornene can guarantee overall better printing performances.

The use of thiol-norbornene chemistry in tomographic printing was first introduced by Rizzo et al. with the cross-linking of photoresins composed of gelatin norbornene (Gel-NB) and thiolated PEG.^70^ In this work, the strain-promoted thiol-norbornene chemistry showed significant improvement in printing time compared to (meth)acrylated counterparts together with excellent cell viability (>95%), thus opening to a broader use of norbornene-modified biopolymers for tomographic bioprinting. Muscle cells embedded in the bioactive and biodegradable photoresin showed cell spreading and proliferation in printed complex 3D models, as well as differentiation into contractile myotubes. Interestingly, the authors also leveraged the fast printing time (10–12 s) to generate a series of perfusable branching constructs, indicating high-throughput printing of tissue models as a promising future direction for tomographic printing (Figure 17D). In a follow-up work, the authors replaced the synthetic thiolated cross-linker with thiol functionalized gelatin (Gel-SH), thus forming a purely naturally derived photoresin.^118^

Besides its use in natural derived photoresins, the norbornene functionality can be applied to a variety of synthetic polymers. For example, Qiu et al. used a photoresin composed of norbornene functionalized PVA (PVA-NB) and a short thiolated PEG cross-linker to print 3D models in the presence of cells.^120^ Unmodified gelatin was added to the formulation to ensure good printability (due to the thermo-reversible gelation of the biopolymer), and, upon thermal removal of gelatin, to obtain soft matrices beneficial for an improved 3D cell growth (Figure 17E). Thanks to the efficient thiol-norbornene chemistry, these matrices were printed within 7–15 s using as little as 1.5% w/v PVA-NB. Thiol-norbornene photoclick chemistry have also been recently exploited by Qiu et al.^120^ and Falandt et al.^157^ to perform photopatterning upon second tomographic projection process. By using a stoichiometric excess of norbornene to thiols, the 3D models printed via tomographic printing feature unreacted norbornene groups that could be used, during a second tomographic printing process, to precisely covalently immobilize thiolated molecules or proteins. The 3D positioning of bioactive molecules and morphogens in biological matrices have been, until recently mostly limited to the lengthy and small-scale (μm-mm) process of two-photon lithography.^182,183^

In another application of tomographic printing, Xie et al. recently reported on the use of a Ru/SPS photoinitiating complex for the printing of pristine silk^76^ (Figure 17F). As discussed in Section 3, the Ru/SPS system can absorb in the visible range (∼400–550 nm) and trigger tyrosine dimerization in a step-growth fashion. Beside the advantage of using longer wavelengths, thus reducing the scattering effect, the Ru/SPS photoinitiator enables the cross-linking of pristine (unmodified) proteins. Although this potentially eliminates the need to chemically modify the starting polymer, it also results in significantly slower gelation kinetics compared to free-radical thiol–ene cross-linking and needs higher polymer concentration due to the generally low abundance of tyrosines when compared to the degree of substitution of polymers modified with thiols and -ene moieties. For example, formulations composed of 2.5%, 5% or 10% silk fibroin and 0.25 mM Ru/2.5 mM SPS all resulted in hydrogels with compressive modulus <500 Pa and a printing time spanning from ∼1 to ∼3 min. To overcome this issue, printed samples were submerged in ethanol. This postprinting treatment is associated with the formation of protein β-sheets, and led to both significant (reversible) volume shrinkage and increase in compressive modulus (from <1 kPa to >200 MPa). The Zhang lab has also recently used the native tyrosine dimerization through the use of Ru/SPS initiator system to tomographically print decellularized extracellular matrix (dECM)-based resins.^75^ Here, the tyrosine groups are prevalent in collagen type I, the primary component of dECM. Using up to 1% of dECM concentration enabled sufficiently high viscosity to prevent part sedimentation and enable successful tomographic printing. In this work, cardiac tissues were created in three dimensions using a special bioink filled with heart muscle cells, which demonstrated positive outcomes in terms of cell growth, cardiac marker expression and coordinated beating. Similarly, knee menisci models were made containing human mesenchymal stem cells, which showed fibrocartilage formation in vitro. This study contributes to the development of a wider range of bioactive photoresins for 3D bioprinting and enhances the application of decellularized extracellular matrix (dECM) in the field of tissue engineering and regenerative medicine.

In light sheet-based printing, Hafa et al. explored thiol-norbornene chemistry for printing skin susbtitutes.^17^ In this study, the primary polymer was composed of norbornene-functionalized dextran with a backbone of PEG (to elongate the chain length), and a thiolated hyaluronic acid was used as the cross-linker. To support cell adhesion, the main precursor was supplemented with cell adhesive RGD (arginyl-glycyl-aspartic acid) sequences, provided by the supplier as RGD-N-Dex, and the hyaluronic acid cross-linker further consisted of a cell-degradable, matrix metalloproteinase-sensitive peptide (CD), also supplied by the manufacturer. In these formulations LAP was used as the cross-linker. Human fibroblasts contained within such thiol–ene chemistry-based gels showed high survival rates (>80% viability). Further, complete skin models showed features of both the dermal and epidermal layers and stayed alive for up to 6 weeks. Their work highlighted the potential of light sheet-based approaches to provide rapid bioprinting of functional tissues along with the ability to monitor the process in real-time.

**Figure 18:**
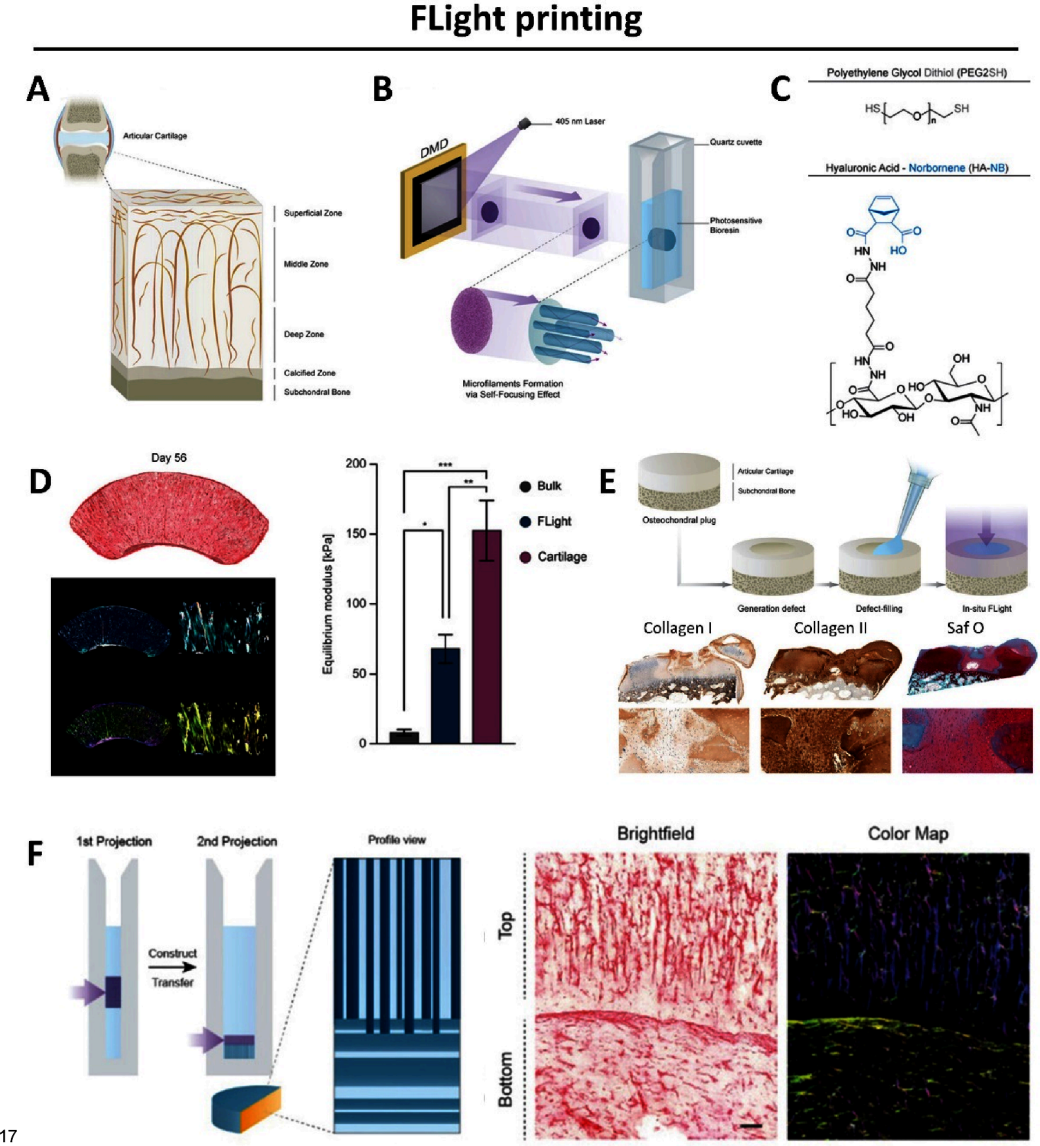
FLight printing of anisotropic cartilaginous constructs using thiol-norbornene based bioresins.^20^**A.** Zonal architecture of an articular cartilage showing the cartilage fiber orientation. B. The cartilage samples were made by filling a glass cuvette with the photoresin, followed by FLight projection. C. The material was based on a thiolated PEG and norbornene-functionalized hyaluronic acid. D. Maturated cartilage constructs (picrosirius red staining (Scale bar: 1 mm)), where the physical guidance provided by the FLight method results in highly aligned collagen fibrils as seen in the polarized light microscopy (PLM) images (Scale bar of full sample: 1 mm; Scale bar of inset: 100 μm). The maturated constructs feature substantially higher equilibrium modulus compared to the samples made using bulk light cross-linking (i.e., without filamentations). E. Schematic illustrating in situ FLight concept, where cartilage constructs were fabricated directly onto bovine knee explants. E. After 56 days of culture, the FLight-filled defects demonstrated anisotropic in situ collagen II deposition, resembling native articular cartilage architecture. F. Schematic of a multiprojection FLight approach to recaptitulate the zone-specific collagen fibril organization of a native articular cartilage. Brightfield image (Picrosirius red staining) and color map of birefringence images acquired by polarized microscopy demonstrate the crisscross organization of collagen in the maturated cartilage constructs. Scale bars: 100 μm. Images reproduced from ref^20^ copyright 2023 Wiley under CC-BY 4.0.

The advent of DVP promises to revolutionize the photopatterning of bioactive signals in cell-laden hydrogels by making it substantially faster and applicable to larger constructs. Interestingly, photoclick chemistry has also recently found use in the biofabrication of articular cartilage using FLight (Figure 18A,B).^20^ A cartilage-tailored photoresin composed of norbornene modified high molecular weight hyaluronic acid and thiolated PEG was used to generate multiple anisotropic, cell instructive constructs in only ∼3 s (Figure 18C). The characteristic aligned microfilaments and microchannels led to directional collagen deposition by the embedded infant polydactyly chondrocytes, resulting in maturation of human cartilage with remarkable native-like mechanical properties, matrix composition and architecture (Figure 18D). The authors also reported on an in situ FLight approach (Figure 18E), leading the way to potentially minimally invasive clinical procedures leveraging FLight projection delivered to the site of damage via optical fibers. In fact, while the other DVP methods rely on relatively complex hardware, optical setup, laser alignment, and software, FLight does not necessitate a highly controlled environment and could be more easily exploited for in vivo/intravital printing. The high modularity of the FLight approach in terms of being able to change the direction of light projection was further used to recapitulate the zonal architecture of the articular cartilage (Figure 18F). In fact, the modularity of the system can also be leveraged to print multimaterial constructs with different filament orientations for different materials (discussed in the next section).^17^

As discussed above, thiol–ene photoresins possess numerous advantages compared to chain-growth counterparts both for biological and nonbiological applications. However, a few drawbacks should be considered.^107,108^ Besides the obvious need of two components (thiolated and ene-modified polymers), thiol–ene photoresins possess a characteristic unpleasant “skunk/rotten eggs” odor, arising especially from volatile short thiolated cross-linkers with low vapor pressure. Although this aspect can be mitigated by the use of high molecular weight thiolated cross-linkers and generally does not represent a significant problem for small scale usage and for the tomographic printing process itself occurring in sealed vials, it might affect thiol–ene usage for large, industrial scale applications where ventilation systems might be necessary. The thiolated components are also responsible for the lower shelf life of such photoresins due to the tendency of disulfide bond formation under oxidative conditions and thermal curing over time. Beside preferring storage of lyophilized/dry polymers and avoiding long-term storage of photoresin solution, shelf life can be prolonged by storage at low temperatures <4 °C, under inert atmosphere, in the dark and further improved with addition of stabilizers (antioxidants, radical scavenging) if compatible with downstream application. The use of Ru/SPS or similar initiating systems (see Norrish Type II, Section 3) is a relevant future research direction, especially for the tissue engineering and bioprinting fields that would significantly benefit from the use of pristine protein-based resins such as collagen, Matrigel, or decellularized ECM. This would guarantee high bioactivity and biomimetic properties for the adopted resins, but also eliminate the need for polymer synthesis or functionalization that often represent an obstacle for the non-expert which slows down the implementation of novel photoresins/chemical strategies for biological applications.

### 5.5 Multimaterial and Hybrid Approaches for DVP

**Figure 19:**
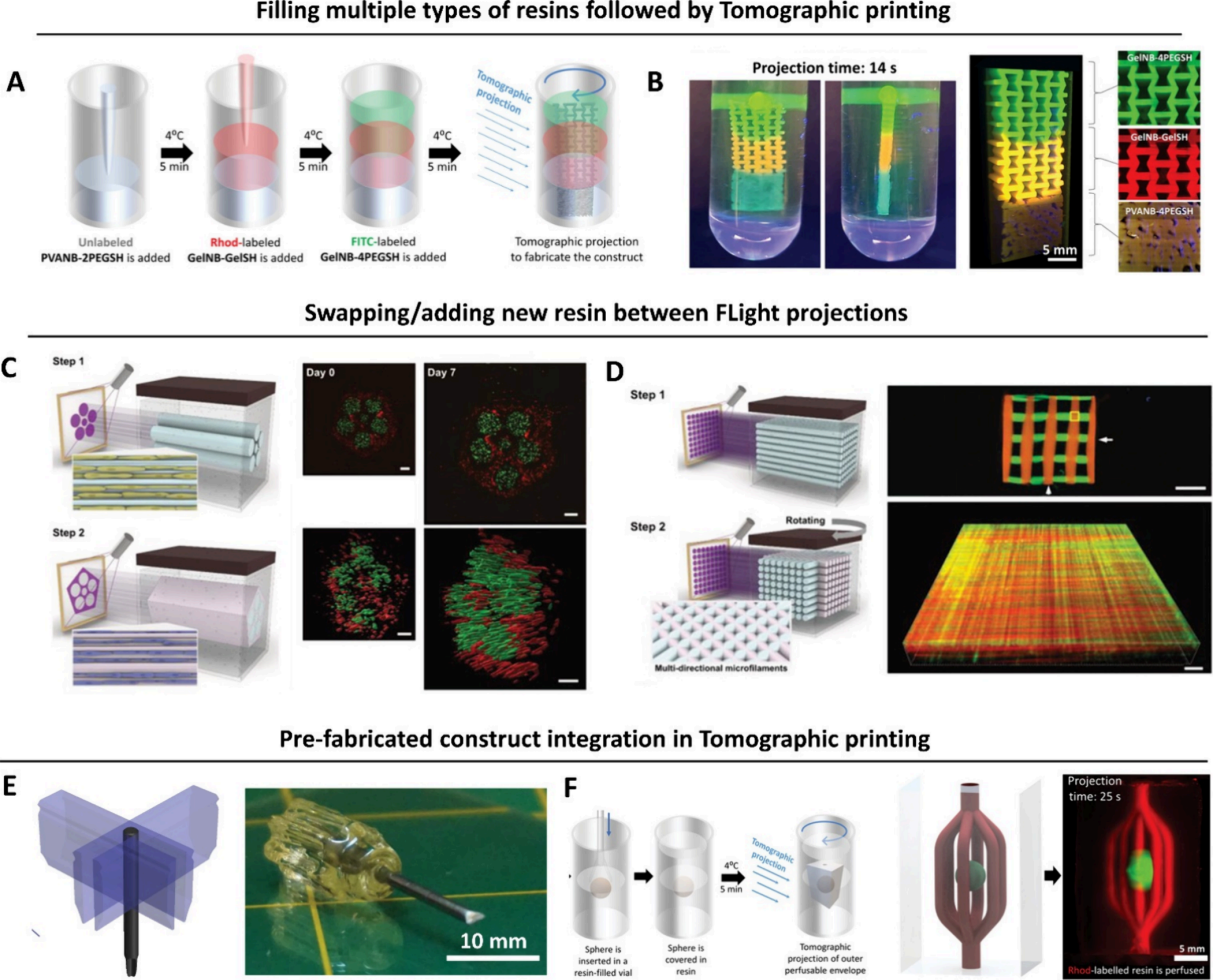
Multimaterial constructs using tomographic or FLight printing. A. Resin swapping scheme for the fabrication of tomographically printed constructs. Here, the photoinitiator concentration should be fine-tuned to be able to match the light dose between the different materials. B. Multimaterial constructs created using a single tomographic projection. Images in panels A and B reproduced from ref^121^ copyright 2023 Wiley under CC-BY 4.0. C. Resin swapping scheme applied in FLight can allow creation of constructs featuring multiple materials across the thickness of the constructs,^44^ the micrographs represent fibroblasts (red) and endothelial cells (green). Scale bars are 200 μm. D. Resin swapping with rotating the vial in-between FLight projection also allows the creation of crisscross microfilament arrangements. Scale bars are 500 μm in the macroscopic and 20 μm in microscopic images, respectively. Images in panels C and D reproduced from ref^44^ copyright 2022 Wiley under CC-BY 4.0. E. Prefabricated part integration approach, where a metal shank is placed within the resin container in the printing zone, resulting in the photo-cross-linked structure to wrap around the prefabricated part (screw driver) (reproduced from ref^41^ copyright 2019 AAAS). F. A prefabricated hydrogel sphere is inserted in a vial partially filled with resin. Subsequent resin filling and tomographic projection fabricates a perfusable construct with sphere encapsulated in the center (reproduced from ref^121^ copyright 2023 Wiley under CC-BY 4.0).

Complex functional requirements of the additively manufactured parts often necessitate the use of multiple materials. A variety of unique processing steps for DVP approaches, as well as hybridization with other manufacturing techniques can enable printing of multimaterial constructs. Perhaps the easiest method involves sequentially filling-in different materials in the printing vial, followed by cross-linking of the entire construct at once. This has been applied to both tomographic^121^ (Figure 19A) and FLight^44^ (Figure 19B) printing techniques. Here, when using different materials requiring different light doses for cross-linking, the photoinitiator concentration can be fine-tuned to allow cross-linking of multiple materials simultaneously.^121^ In this technique, resin viscosity should be sufficiently high to ensure that the different constructs do not mix, unless such mixing maybe desired (e.g., for tissue interfaces^184^). Another technique involves anchoring the first material construct within the printing vial, followed by switching the un-cross-linked resin with a different material composition and proceed to tomographic projection print the subsequent layer. This approach has been applied in tomographic^121^ and FLight^44^ approaches (Figure 19C,D). In particular, the tomographic printing approach requires removal of the suspending beams after printing, which may render the process tedious.^121^ Also, the refractive index of the materials will need to be matched (e.g., by using Iodixanol) to allow minimal light scattering due to an already-cross-linked layer when printing the next layer.^121^ The third approach integrates a prefabricated construct into a resin-filled container, followed by projecting the light to cross-link the resin around the construct^41,121^ (Figure 19E,F). This approach could be desirable, in that one or more materials could be processed using a conventional manufacturing approach, while the tomographic printing is done across the prefabricated construct^41,121,37^.

**Figure 20:**
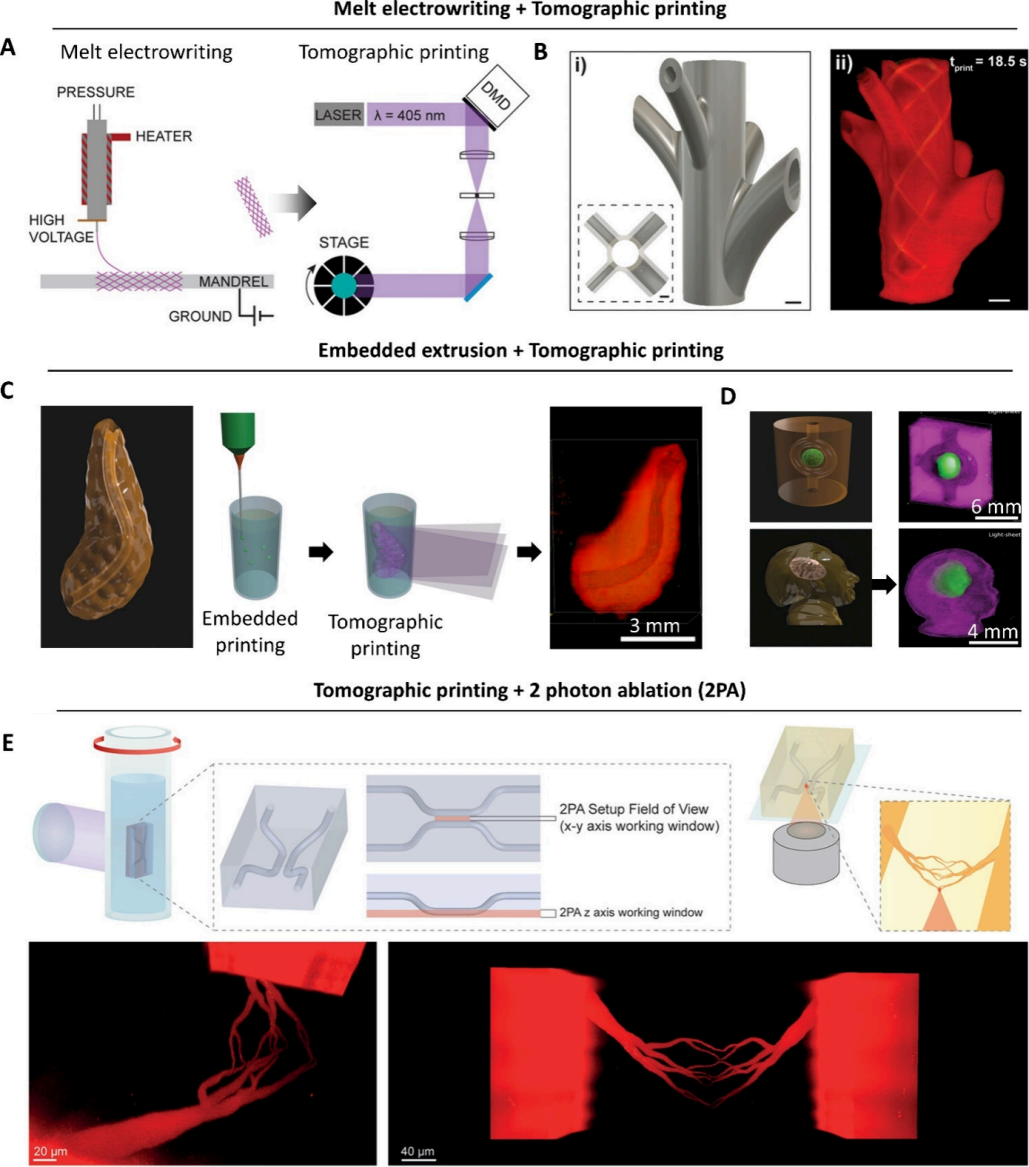
Hybrid approaches for tomographic printing. A. Hybridization of melt electrowriting and tomographic printing, where a melt-electrowritten tube is inserted and localized within a vial, followed by tomographic projections to cross-link hydrogel structures around the melt-electrowritten construct.^146^ B. Example print of a branched vascular graft featuring melt electrowritten PCL tube and a tomographically printed GelMA vessel structure. Scale bar is 1 mm. Images from A and B reproduced from ref^146^ copyright 2023 Wiley under CC-BY 4.0. C. Hybrid process of embedded extrusion and tomographic printing,^150^ where different material components are added to the printing vial using embedded printing, followed by tomographic light projections to fabricate the multimaterial constructs (panel D shows some selected constructs) (reproduced from ref^150^ copyright 2023 Wiley under CC-BY 4.0). E. Hybrid process of tomographic printing and two-photon ablation (2PA),^159^ where tomographic printing is used to cross-link hydrogel networks featuring large-scale vessels, and 2PA has been used to print small diameter (φ ∼ 2 μm) perfusable microvessels in-between (reproduced from ref^159^ copyright 2023 Wiley under CC-BY 4.0).

While the methods described above can allow substantial freedom in printing complex multimaterial structures, the intrinsic limitation of resolution of DVP (minimum feature sizes typically ≥25 μm), may necessitate the hybridization of DVP techniques with other fabrication processes. For instance, Größbacher et al.^146^ integrated melt electrowritten polycaprolactone (PCL) constructs within photoresin-containing vials, followed by tomographic projections to fabricate hydrogel constructs integrated within and around the PCL constructs (Figure 20A). The constructs featured improved mechanical properties (e.g., Young’s modulus and tensile strength) and performance (e.g., burst pressure in vessel constructs) than their single material counterparts. In addition to leak proofing, this approach allowed the creation of complex structures such as bifurcating vessels which could enhance the clinical applicability of the melt electro-written grafts (Figure 20B). In another approach, Ribezzi et al.^150^ used embedded printing to position different material components (e.g., cellular spheroids in Figure 20C,D) within a printing vial, followed by tomographic projections to fabricate the cell-laden structures with perfusable channels. Notably, GelMA microgels in the extrusion printing acted as a support bath for the embedded extrusion printing, while tomographically cross-linking the constructs allowed the retention of interstitial microvoids within the constructs which allowed better nutrient transport and tissue maturation in the constructs compared to bulk hydrogels. A multiscale and high-resolution perfusable system was demonstrated by Rizzo et al., who fabricated macro-channeled constructs using tomographic printing, and then used two photon ablation to create perfusable microchannels (Figure 20E) as small as 2 μm.^159^ Very recently, Riffe et al. from the Burdick lab demonstrated that photo-cross-linkable gelatin and hyaluronic acid containing suspension baths can enable facile extrusion printing of multimaterial resins, and the whole bath could later be processed for tomographic printing to create complex multimaterials constructs.^123^ While hybrid approaches represent a promising avenue for future research in multimaterial DVP, it is important to consider different materials being printed may need different light doses for printing. Furthermore, the light may deviate or scatter of due to differences in refractive indices of the materials. Here, light dose matching by changing the photoinitiator concentration in the different materials, or refractive index matching as described above can help minimize light scattering and maximize resolution.^121^

## 6 Future Scope for Improvement in DVP

As with any fabrication methods, DVP approaches offer several future research opportunities to improve the capabilities of the different processes in terms of printing larger objects faster and at a higher resolution, with possibilities of resin reuse. These have been discussed below.

### 6.1 Scaling Up the Build Volume

Scaling up the build volume to several centimeters can allow the creation of anatomically relevant constructs, 1:1 scale prototypes and also ready-to-use functional objects. From an optical standpoint, changing the image magnification after DMD can enable printing larger structures. After light shaping through the DMD, the image is projected into a 4f lens system, which usually consists of a pair of two plano convex lenses. Here, the first lens captures the image reflected from the DMD, and focuses it onto a single point in-between the lenses. The other lens then captures the focused image and then collimates it such that it can be projected into the resin vat. There is usually an iris kept at the focal point of the first lens (i.e., the lens near the DMD) such that the auxiliary images generated from the DMD can be removed. Here, depending on the focal length of the lenses, the image from the DMD can either be magnified or reduced. The image magnification from a 4f system defined as f2/f1, where f1 is the focal length of lens near the DMD and f2 is the focal length of the lens near the printing vial.^185^ Accordingly, larger f2 would enable larger prints, albeit with a loss in resolution and fine features of the images. Here, DMDs featuring smaller pitch (higher resolution) can be used to circumvent the loss in resolution due to image expansion. For instance, a DMD featuring a 4k resolution, would still allow an image which is expanded 4 times to feature a resolution close to 1080p.

As the build volume is enlarged, light attenuation will increase (eq 2), which would require reducing the concentration of photoinitiating species in the photoresin, which in turn would reduce the reactivity of the resin and lengthen the fabrication time for larger constructs. Here, step-growth cross-linking can allow faster prints by enabling a more rapid light response and photo-cross-linking, which can prevent nonspecific cross-linking of the constructs. Leveraging the near-infrared (NIR) optical window in DVP could also help to extend the range of light penetration and photo-cross-linking in the build volume.^124^ Efficient red-shifted photoinitiator compounds or the use of up-conversion nanoparticles are promising areas of exploration.^111,186^

Ingenious process design can also enable larger prints. For instance, larger prints in tomographic printing have been made possible by introducing a helical movement mechanism,^152^ which allows indexing the vial position between tomographic projections to enable printing of constructs three times as large as those without a helical movement. One can envision such a system being controlled with a robotic arm, which could not only perform helical motions, but also print within multiple vials or within different regions of a single vial. For FLight printing, to obtain larger constructs, the light attenuation due to absorption and scattering limits the formation of longer constructs. Here, one option is to use longer wavelengths of light (with the appropriate photo-cross-linking chemistry) to achieve higher penetration and lower attenuation in the resin. Of course, the absorption coefficient of the photoinitiator will also have to be accounted for, as a highly absorbent photoinitiator with absorption maxima at higher wavelengths (see Figure 8) will not allow light penetration deep into the photoresin. In terms of cross-linking chemistry, deploying step-growth polymerization can also allow faster cross-linking and less time for free radical diffusion, which could further allow the fabrication of longer constructs without causing undesired material cross-linking. In terms of a hybridization scheme for FLight printing, a top-down projection of FLight can be synchronized with continuous resin feeding in a container. Here, addition of photo absorbers^5,187^ may be necessary to limit excessive light dose in the layers which have already been photo-cross-linked. To create physiological-sized hydrogel constructs with aligned filaments, this approach can leverage the precise control of light penetration depth and photoresin flow, ensuring that while the macro-level constructs are adequately photo-cross-linked, the internal microstructure still has the microscale configuration induced by the self-focusing effect. Similarly, these principles are applicable to light sheet-based stereolithography; when combined with the continuous feed of resin, the creation of extensive and coherent structures is possible.^21^

### 6.2 Improving Spatial Printing Resolution

Section 4 highlighted current procedures for improving the spatial printing resolution. In DVP techniques, while process hybridization can overcome the limitation of resolutions, it is often tedious to integrate different types of processes, and each process presents its own limitations. For instance, two photon ablation is unable to ablate structures more than a few mm in depth and melt electrowriting is also limited in terms of the structural complexity and fiber orientations it can achieve. Here, there are several considerations to improve the printing resolution of individual DVP processes. The first consideration is through changing the way the light is projected into the photoresin. The current DVP landscape is dominated by the use of continuous wave light sources, and the overlap of light scattering with free radical diffusion can lead to cross-linking of undesired areas, which in turn affects the printing fidelity. A relatively facile method to achieve higher print resolution involves pulse width modulation (PWM) of the projected light.^188,189^ In these approaches, light is administered in brief bursts of milliseconds, rather than as a continuous exposure. When exposed to these flashes, the prepolymer substance barely scatters the light, which would otherwise occur due to changes in the refractive index of the polymer through gradual cross-linking. Subsequently, in the absence of light, the material undergoes polymerization. As a result, the pattern of light exposure remains minimally affected by scattering.^188,189^ For determining the optimal duration between light flashes, You et al.^188^ hypothesized that, as the free radical lifetime is typically around 10 ms, a flashing dose of a few milliseconds duration but with higher light intensity can allow higher fidelity prints compared to exposure over a few seconds. Notably, as the light is projected into the whole vat in DVP approaches, there is a possibility that such a high light dose could initiate cross-linking in regions other than the printing zone. As such, careful optimization of the light dose will be needed to achieve rapid and high fidelity DVP using flashing polymerization.

Another important consideration for achieving high resolution prints is reduction of speckle noise from the coherent light sources within Deep Vat techniques.^191^ Notably, speckle noise is important for the emergence of microfilaments within FLight technique.^44^ However, for other Deep Vat techniques, speckle noises can reduce the sharpness and resolution of laser-based projections and imaging,^190^ as well as the uniformity of illumination distribution.^117^ Optical components and add-ons, such as vibrating multimode fiber bundles,^191^ multiscattering particles (colloidal dispersion), and diffusers,^192^ have been found to effectively reduce speckle noise.

Specific additives like nanofillers^193^ or refractive index matching^49,194^ can also reduce light scattering, enhancing linear light transmission, to achieve higher resolution and enable larger build volumes. These additives can also be used to adjust the photoresin’s mechanical properties and curing characteristics to meet diverse printing requirements. Adding free radical quenching agents such as TEMPO to the photoresin can help in preventing nonspecific cross-linking.^23,54^ These inhibitors or quenchers can be designed to react with free radicals or cations generated during the photo-cross-linking, effectively stopping the reaction from progressing in unintended areas. They can be particularly useful in thin boundary layers where light might inadvertently cause unwanted cross-linking. Furthermore, in the presence of scattering particles such as cells, as has been extensively demonstrated, refractive index matching can also prevent unwanted light scattering and improve print resolution.^49,159^

The combination of machine learning and computational modeling has already been utilized in improving print resolution in additive manufacturing processes.^195,196^ This includes the prediction of polymer network formation and its kinetics, light propagation/attenuation and heat generation, and design iterations optimized for different applications. Similarly, machine learning shows promise to improve the resolution of the DVP using models trained with properties of resin additives, monomers, and cross-linking through a cross-disciplinary training database (chemistry-physics-engineering).

### 6.3 New Photoresin Formulations

In Section 3, we have highlighted several photoresin formulations and photoinitiating systems that have not been explored in the field of DVP. In Section 2, we have discussed the advantages and limitations of chain-growth and step-growth-based photoresins. Of these, chain-growth resins are more widely explored for the different biomedical and nonbiomedical application so far. For step-growth cross-linking systems, it is reasonable to expect in the near future an increasing number of studies investigating DVP with a wide variety of NB-modified biopolymers such as collagen, chitosan,^197^ hyaluronic acid,^57,198−200^ alginate,^201,202^ dextran,^203^ and silk,^204,205^ to name a few, for which synthesis protocols are already available in literature. Similarly, the use of the NB moiety is expected to benefit processing of materials for nonbiological applications. The use of the Ru/SPS initiating system is also expected to play a central role in bioprinting in the near future, thanks to its visible-light absorption, commercial availability, and the possibility to be used with unmodified, tyrosine rich photoresins. In particular, it is important to highlight that dityrosines are also formed under oxidative conditions in native tissues, making this cross-linking inherently different than those using non-natural moieties (i.e., methacryloyl, norbornene). It is also important to consider that being in its infancy, the field of DVP has not yet explored a wide variety of efficient photoclick strategies such as thiol–yne,^206−208^ photo-SPAAC,^209,210^ tetrazole-ene cycloaddition,^211−213^ tetrazine ligation,^214−216^ and other formulations highlighted by Fairbanks and colleagues.^71^

In terms of the photoinitiation system, we have highlighted the photoinitiators which have not yet been explored for DVP approaches (Figure 8). We also previously highlighted that red-shifted photoinitiators represent a promising advance in the DVP field, allowing deeper light penetration and larger build volumes. Here, we have highlighted photoredox catalysts based on xanthene dyes, BODIPY cores^92^ and metal complexes^93^ to produce initiating radical species with long wavelengths. Notably, the limited water solubility of these systems and the potential cytotoxic effects of the electron transfer processes is another scope of improvement which needs to be tackled to improve their usage in DVP.

Regardless of the type of photo-cross-linking mechanism, one of the major challenges with DVP techniques is related to the reusability of the un-cross-linked portion of the resin. Considering that the whole vat can be exposed to light doses below the gelation threshold, it is reasonable to assume that photochemical reactions occur everywhere in the vat to a certain degree, thus changing the state of the polymeric solution (i.e., formation of branched polymers and kinetic chains outside of the cross-linked network, consumption of the photoinitiators). This aspect is particularly relevant for the printing of large volumes and industrial/high throughput applications, where un-cross-linked resin can represent a major source of material waste and expenses. Photochemical strategies that can lead to full recyclability of un-cross-linked parts would therefore be highly beneficial for the field of DVP. Besides, the container sizes for DVP applications should be judiciously chosen based on the size of the printed constructs to minimize resin wastage. For bioprinting applications, the limited availability and expensive processing of cells also necessitates the reuse of the resin. However, the cells within the reused resins may be exposed to higher light doses than the cells in the constructs which were fabricated prior. This may affect the cell viability and may lead to undesired changes in the gene expression of the cells, thereby reducing the reusability of the resin. Future studies should also investigate the reusability of cell-laden resins and the optimal light wavelength and exposure which enables similar attributes (viability, gene and protein expression, tissue maturation) of the printed cell-laden constructs during the different iterations of printing.

### 6.4 New Approaches for Printing Multimaterial Constructs

Printing constructs featuring multiple material constitutions with controlled spatial organization is an essential step in the evolution of DVP and is critical to enable the broad-spectrum utilization in biomedical and other industrial fields. Notably, there have been attempts with tomographic and FLight printing as standalone process (Figure 15), or in combination with hybrid approaches (Figure 16) to print multimaterial structures. However, to date, light sheet-based printing has not been hybridized nor shown with multiple materials, representing another promising area of exploration.

To address the challenges of working with different materials in printing, one potential solution is to use a single resin composition that allows the constituent materials to cross-link under different wavelengths. This would enable the printing of multimaterial constructs without the need to replace resins between different prints or add multiple resins within the print chamber. For instance, Wang et al. used a single material capable of being tomographically printed using multiple wavelengths for stiffness control within the constructs.^42^ In this approach, the wavelength-sensitive photoresins were cured using a visible (455 nm) and UV (365 nm) light source simultaneously, thus providing spatial control over material stiffness. The introduction of the epoxy monomers to the acrylate resin altered the nonlinearity in its photoresponse while leaving the cross-linking threshold unaffected.^42^ A similar technique could be adapted to cross-link different material constituents within a single resin by employing different wavelengths for tomographic projection. In this scenario, non-cross-linked constituents can simply diffuse away after printing. However, optimizing the dosage for printing different constituents and assessing the impact of multiple photoinitiator species on cells may pose challenges that need further characterization.

## 7 Concluding Remarks

DVP techniques are rapidly evolving, with each approach having distinct advantages and challenges, and offering a unique path to creating complex structures. Compared to tomographic printing, light sheet and FLight printing approaches are relatively new and underexplored. Nevertheless, each technique offers tremendous potential for exploring new avenues for research in the biomedical and nonbiomedical sectors. Here, we highlighted the commonalities and differences in process principles between the different techniques and resin formulations. We hope this review article will find interest among the chemists, physicists, biologists, material scientists, engineers and clinicians who would like to (i) Utilize the most appropriate fabrication approach for their application and materials, (ii) understand the critical photoprocessing and materials constraints in DVP approaches, (iii) rationally tune the materials and photoresins for compatibility with the DVP, (iv) develop new hybrid approaches or more advanced Deep Vat approaches for adding complexity to the prints, and (v) explore new applications areas for DVP techniques. Notably, due to the tremendous rate of advances in the field of DVP, we anticipate that Deep Vat techniques will soon be on par with conventional stereolithography approaches in terms of the process capabilities, speed, and efficiency.
